# Hemoglobin in the brain frontal lobe tissue of patients with Alzheimer’s disease is susceptible to reactive nitrogen species-mediated oxidative damage

**DOI:** 10.1016/j.redox.2025.103612

**Published:** 2025-03-25

**Authors:** M.J. Smallwood, M. Abu Alghayth, A.R. Knight, K. Tveen-Jensen, A.R. Pitt, C.M. Spickett, D. Llewellyn, G. Pula, A. Wearn, A. Vanhatalo, A.M. Jones, P. Francis, E. Coulthard, P.G. Kehoe, P.G. Winyard

**Affiliations:** a University of Exeter Medical School, Exeter, EX1 2LU, UK; b College of Health & Life Sciences, Aston University, Birmingham, B4 7ET, UK; c Centre for Biomedicine, Hull York Medical School, Hull, HU6 7RX, UK; d Translational Health Sciences, Bristol Medical School, University of Bristol, Southmead Hospital, Bristol, BS10 5NB, UK; e Institute of Psychiatry, Psychology and Neuroscience, King’s College London, London, WC2R 2LS, UK; f Current Address: Department of Medical Laboratory Sciences, College of Applied Medical Sciences, University of Bisha, Bisha, P.O. Box 255, 67714, Saudi Arabia

**Keywords:** Alzheimer’s disease, Vascular dementia, Nitrative stress, 3-Nitrotyrosine, Hemoglobin, Frontal lobe brain tissue

## Abstract

Brain inflammation in Alzheimer’s disease (AD) involves reactive nitrogen species (RNS) generation. Protein contents of 3-nitrotyrosine, a product of RNS generation, were assessed in frontal lobe brain homogenates from patients with AD, patients with vascular dementia (VaD) and non-dementia (ND) controls. Western blotting revealed a dominant 15 kDa nitrated protein band in both dementia (AD/VaD) and ND frontal lobe brain tissue. Surprisingly, this protein band was identified by mass spectrometry as hemoglobin, an erythrocytic protein. The same band stained positively when western blotted using an anti-hemoglobin antibody. On western blots, the median (IQR) normalized staining intensity for 3-nitrotyrosine in hemoglobin was increased in both AD [1.71 (1.20–3.05) AU] and VaD [1.50 (0.59–3.04) AU] brain tissue compared to ND controls [0.41 (0.09–0.75) AU] (Mann-Whitney *U* test: AD v ND, P < 0.0005; VaD v ND, P < 0.05; n = 11). The median normalized staining of the nitrated hemoglobin band was higher in advanced AD patients compared with early-stage AD (P < 0.005). The median brain tissue NO_2_^−^ levels (nmol/mg protein) were significantly higher in AD samples than in ND controls (P < 0.05). Image analysis of western blots of lysates from peripheral blood erythrocytes suggested that hemoglobin nitration was increased in AD compared to ND (P < 0.05; n = 4 in each group). Total protein-associated 3-nitrotyrosine was measured by an electrochemiluminescence-based immunosorbent assay, but showed no statistically significant differences between AD, VaD and ND. Females showed larger increases in hemoglobin nitration and NO_2_^−^ levels between disease and control groups compared to males, although the group sizes in these sub-analyses were small. In conclusion, the extent of hemoglobin nitration was increased in AD and VaD brain frontal lobe tissue compared with ND. We propose that reactive nitrogen species-mediated damage to hemoglobin may be involved in the pathogenesis of AD.

## Highlights

• Western blots revealed a 3-nitrotyrosine-containing 15 kDa protein band in human frontal lobe brain tissue lysates.
• Surprisingly, the 15 kDa protein was identified by mass spectrometry and immunoblotting as hemoglobin.
• Brain hemoglobin nitration was increased in Alzheimer’s disease and vascular dementia compared to non-dementia tissue.
• Hemoglobin nitration was higher in circulating erythrocytes from Alzheimer’s disease patients compared to non-dementia cells.
• Reactive nitrogen species-mediated oxidative damage to hemoglobin occurs in Alzheimer’s disease.

## Graphical abstract

**Figure:**
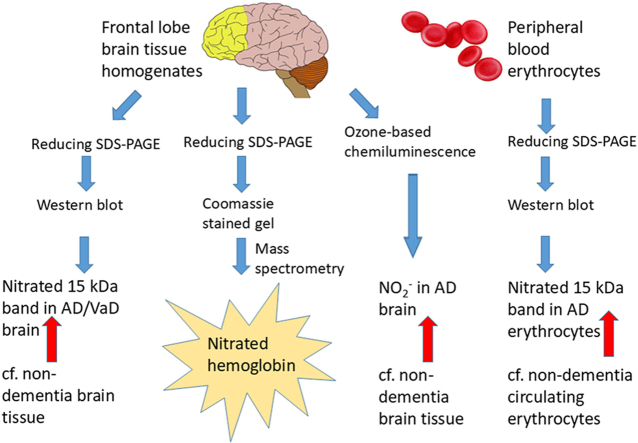

## 1 Introduction

As human life expectancy increases, so do the health care consequences of an ageing population, one of which is an increasing prevalence of dementia [1]. The prevalence of dementia has been estimated to increase from 57·4 million cases globally in 2019 to 152·8 million cases in 2050 [2]. Alzheimer’s disease (AD), a sub-type of dementia makes up approximately 70 % of all dementia patients [1]. The second largest sub-type, vascular dementia (VaD), (increasingly known as Vascular Cognitive Impairment (VCI) [3], is characterized by hypoxia due to reduced cerebral blood flow or infarction [4]. However, the clinical reality is that a ‘mixed’ dementia (AD with cerebrovascular disease) sub-type (or indeed other sub-types of dementia with vascular pathologies) is often underestimated in the patient population [5]. Vascular dysregulation may be one of the earliest events in the evolution of AD, preceding the classical amyloid- and tau-related hallmarks of AD pathology [6]. Similarly neuro-inflammation, which can be promoted by mediators of both vascular dysfunction and pathology in AD [7,8] is an important factor in AD progression [9]. Resident innate immune cells (microglia and astrocytes) within the central nervous system are activated by amyloid plaques, while neurofibrillary tangles [10] and plaques activate complement pathways [11].

Oxidative and nitrative stress are common in dementia [[12], [13], [14], [15]] and result in the generation of increased levels of nitrated proteins (such as proteins containing 3-nitrotyrosine residues), protein carbonyls, malondialdehyde (a product of lipid peroxidation) and advanced glycation end products (AGEs). Oxidative damage to enzymes involved in glycolysis, the tricarboxylic acid cycle and ATP biosynthesis, contributes to decreased glucose metabolism [15]. Amyloid-β and tau both trigger mitochondrial alterations that are involved in synaptic dysfunction, synaptic loss and neuronal death which manifest themselves in impaired cognitive function [16]. During normal ageing, neurons progressively accumulate somatic mutations [17] and somatic neuronal DNA alterations due to nucleotide oxidation were found to be increased in AD patients compared with control individuals [18], implicating oxidative stress in the pathogenesis of AD. Oxidative/nitrative stress pathways which may play a role in brain tissue damage include: (a) the generation of reactive oxygen species, e.g. hydrogen peroxide (H_2_O_2_), induced by amyloid-β plaques [[19], [20], [21]]; (b) amyloid-β-induced nitric oxide (NO) synthase expression leading to increased NO production [22]; (c) NO upregulating the expression of heme oxygenase-1, which mediates the degradation of heme to ferrous iron and is associated with neurofibrillary pathology [23] and (d) increased expression of the heme enzyme, myeloperoxidase (MPO) [24,25].

These vascular and inflammatory mediators may interact within the biochemical environment of the AD brain [26], thereby producing a nitrating system composed of MPO, H_2_O_2_ and NO_2_^−^, that is induced in AD [25,26]. *In vivo*, NO is unstable and is quickly oxidized to the stable metabolites, nitrite (NO_2_^−^) and nitrate (NO_3_^−^). One proposed pathway [27] for the nitration of tyrosine to form 3-nitrotyrosine is shown in equations (1), (2), (3), (4)):

(1) H_2_O_2_ + MPO → Compound I + H_2_O

(2) NO_2_^−^ + Compound I → Compound II + ^•^NO_2_

(3) Compound I + Tyrosine → Compound II + Tyrosine^•^

(4) Tyrosine^•^ + ^•^NO_2_ → 3-Nitrotyrosine

The above system has the capacity to nitrate tyrosine residues within proteins, generating post-translationally modified proteins containing 3-nitrotyrosine residues. Protein nitration may also occur via the rapid reaction of NO with superoxide, O_2_^**.**^^−^, to generate the potent nitrating agent, peroxynitrite (ONOO^−^) [27]. Nitrated proteins have been observed in human AD brain tissue [[28], [29], [30], [31], [32], [33], [34], [35]] and cerebrospinal fluid (CSF) [36]. Total protein 3-nitrotyrosine has been shown to be increased in the inferior parietal lobule and hippocampus brain regions in amnestic MCI patients compared to those regions from control subjects [34,37]. Increased 3-nitrotyrosine was also observed in the medial temporal lobe (including the hippocampus) and orbitofrontal cortex [29], in the inferior parietal lobule [32,38], and in the hippocampus and cerebellum [39,40] from patients with AD. MPO is increased in hippocampal neurons [25] and a functional polymorphism in the MPO gene was associated with AD [24]. Oxidative stress and H_2_O_2_ are implicated in the neurotoxicity of amyloid-β [19,41], while amyloid-β oligomers generate H_2_O_2_ [21]. Nitrated proteins in the AD brain include α-enolase, triose phosphate isomerase, ATP synthase, voltage-dependent anion channel protein 1 and β-actin [42]. Furthermore, nitrated tau was observed in neurofibrillary tangles, with evidence that tau nitration occurred early in the disease [43].

We hypothesized that there are increased levels of NO_2_^−^, NO_3_^−^ and nitrated proteins within the frontal lobe brain tissue of AD and VaD patients compared to non-dementia (ND) controls. We therefore set out to detect these entities in brain tissue. We also tested whether the levels of nitrated proteins were correlated with pathological disease severity, as assessed by the Braak classification, a staging system used to classify the degree of pathology in AD [44].

## 2 Methods

### 2.1 Patient and control samples

Human brain tissue was obtained from the Human Tissue Authority licensed (license number 12273) “South West Dementia Brain Bank” (SWDBB), University of Bristol, UK, with local research ethics committee approval (National Research Ethics Service 08/H0106/28 + 5) and informed consent from participants in the study. Neuropathological examination was undertaken by a neuropathologist. Control brain tissue samples were from people who had no history of dementia, had been extensively assessed neuropathologically, and had few or absent neuritic plaques, a Braak tangle stage 3 or less, and no other neuropathological abnormalities [45,46]. AD brains had detailed neuropathological assessment according to the National Institute on Aging-Alzheimer’s Association guidelines [47], and AD pathology was a sufficient explanation for the dementia in these cases. Human brain tissue was collected from the left midfrontal lobe (an area of brain affected early on in AD [48]) of frozen hemispheres from 15 AD patients, 15 VaD patients and 15 non-dementia (ND) controls patients. The demographics of the patients and control individuals are summarised in Table 1. Equivalent areas of brain tissue, approximately 200 mg/ml in cold lysis buffer (1 % SDS, 100 mM NaCl, 10 mM Tris(hydroxymethyl)methylamine; pH 7.6, 1 mg/ml aprotinin, 1 mg/ml phenylmethanesulfonyl fluoride) were homogenized in a Precellys 24 Homogenizer (Stretton Scientific Ltd, Derbyshire UK) using 2.3 mm ceramic beads. The samples were homogenized at 3000 *g* for 15 s, then left for 3 min on ice before the process was repeated. The samples were centrifuged at 14,000 *g* for 10 min and the supernatants aliquoted and stored at −80 °C until analysis. The AD group was selected for brain tissue analysis on the basis of a diagnosis according to the “Consortium to Establish a Registry for Alzheimer’s Disease” (CERAD) criteria of “definite AD” [49]. The recruited AD patients had a Braak tangle stage of 4–6. Braak stages 1 and 2 are characterized by neurofibrillary tangle involvement that is confined mainly to the transentorhinal region of the brain; stages 3 and 4 are characterized by the additional involvement of limbic regions such as the hippocampus; and stages 5 and 6 are characterized by extensive neocortical involvement [50]. VaD cases had a clinical history of dementia, occasional neuritic plaques (if present), a Braak tangle stage of 3 or less, histopathological evidence of multiple infarcts/ischemic lesions, moderate to severe atheroma and/or arteriosclerosis, and an absence of histopathological evidence of other disease likely to cause dementia.

**Table 1:** Summary of demographics of control, VaD and AD cases used for brain sample analysis.

	Age-at-death	Gender	Postmortem	Braak tangle stage		
	(years ± SD)	(F:M)	delay (h ± SD)	0-II	III-IV	V-VI
Controls (n = 15)	79.2 ± 11.6	6:9	43.2 ± 29.5	11	4	0
VaD (n = 15)	81.9 ± 9.1	7:8	44.9 ± 20.9	10	5	0
AD (n = 15)	77.4 ± 9.2	10:5	43.2 ± 29.5	0	0	15

Human peripheral blood erythrocyte samples were collected from AD patients and ND volunteers who attended North Bristol NHS Trust, Southmead Hospital, Bristol UK. Participants were recruited from North Bristol Trust NHS cognitive disorders clinic, local volunteer databases, the Join Dementia Research platform and word of mouth. All participants were verbally screened for a history of neurological disorders and memory problems in a telephone interview. All patients provided informed written consent prior to testing. Ethical approval was given by Frenchay NHS Research Ethics Committee. Erythrocytes were stored at −80^o^C until analysis. Defrosted samples of lysed erythrocytes were centrifuged at 14,000 *g* for 15 min to remove cellular debris.

### 2.2 Measurement of brain amyloid-β load

Paraffin sections 7 μm in thickness were cut from blocks of the brain frontal lobes. After pretreatment for 20 min in 100 % formic acid and subsequent blocking in horse serum solution, the sections were incubated overnight at room temperature with antibody to Aβ (1:2000, DAKO M0872, raised against Aβ residues 8–17, Vectalabs, Peterborough, UK). Bound antibody was visualized by incubation with biotinylated Universal Antibody (Vectastain Universal Elite; Dako, Ely, Cambridgeshire, UK) and visualized with avidin-biotin horseradish peroxidase complex kit from Vector Laboratories, Burlingame, CA, USA). Histometrix software (Kinetic Imaging, Wirral, UK) driving a Leica DM microscope with a motorised stage was used to make an unbiased selection of the 10 areas, as previously described [51]. Aβ-laden blood vessels were excluded from analysis.

### 2.3 Peroxynitrite synthesis, and nitration of bovine serum albumin and human hemoglobin for use as positive control samples

Nitrated bovine serum albumin (BSA) and nitrated human hemoglobin (isolated from blood) were prepared from the corresponding native proteins (both from Sigma Aldrich, Gillingham, Dorset, UK) for experiments where nitrated albumin and/or nitrated hemoglobin was required as a positive control to confirm binding of the anti-nitrotyrosine antibody. ONOO^−^ was first synthesized as a nitrating agent according to the method described by Beckman et al. (1994a). In ice-cold glassware, acidified H_2_O_2_ (0.7 M, 0.6 M HCl) was quickly mixed with NaNO_2_ (0.6 M), and this was immediately followed by the addition of NaOH (1.2 M), resulting in a yellow solution of diluted ONOO^−^. MnO_2_ (∼15 mg) was added to deplete excess H_2_O_2_, and the solution was then filtered and frozen at −20 °C. The top layer of concentrated ONOO^−^ thaws most rapidly. This layer was collected, and the ONOO^−^ concentration was calculated from the absorbance at 302 nm (extinction coefficient 1670 M^−1^ cm^−1^ (Hughes and Nicklin, 1968). For the nitration of BSA or hemoglobin, solutions of 2 mg/ml protein in bicarbonate-based nitration buffer (potassium dihydrogen phosphate (100 mM) and sodium bicarbonate (25 mM)) were mixed with ONOO^−^ (3 mM), whilst being gently vortexed. Nitration was measured, at pH 10, on a Cary 300 UV–vis spectrophotometer (Agilent Technologies LDA UK Limited, Stockport, Cheshire UK), using the extinction coefficient at 428 nm of 4200 M^−1^ cm^−1^ (van der Zee et al., 1977). For some experiments, where indicated, nitrated BSA was purchased from Enzo Life Sciences (Exeter, Devon, UK).

### 2.4 Western blotting of nitrated proteins from human brain and peripheral blood erythrocytes

For reducing SDS PAGE, FastCast acrylamide kits (12 % gels) or Mini-protean plates (8–16 % gradient gels), nitrocellulose membrane (0.45 μm), and transfer packs were from Bio-Rad (Hemel Hempstead, Hertfordshire, UK) and were used according to the manufacturer’s instructions. The gel percentage in each experiment is detailed in each figure legend. Two samples from each group (ND, VaD and AD) were run per gel, with 60 μg of protein per lane and 2-mercaptoethanol used as the reducing agent and samples were heated to 99^o^C for 5 min. β-Actin or hemoglobin were used as the loading controls (as indicated in figure legends) and nitrated BSA was used as a positive control, to verify binding of the anti-nitrotyrosine antibody. Transfer from the SDS-polyacrylamide gel to the membrane (nitrocellulose, 0.45 μm, Bio-Rad) was performed using the ‘wet’ tank electro-transfer method, using a constant current of 0.35 A for 60 min (Bio-Rad). For the probing of western blots of brain tissue extracts, the membrane was blocked with protein-free blocking buffer, 60 min at room temperature. Mouse monoclonal anti-nitrotyrosine and rabbit polyclonal anti-β actin (human) antibodies (product numbers 189542 and A300-485A respectively, Cambridge Biosciences, Cambridge, UK) were diluted 1:1000 in protein free blocking buffer with 0.05 % Tween and incubated for 1 h at room temperature. A near infra-red fluorescence-labelled secondary anti-mouse antibody (IRDye800CW, green fluorescence, 925–32210, Li-Cor Biosciences, Cambridge, UK) was used for detection of 3-nitrotyrosine. A secondary anti-rabbit antibody (IRDye680RD, red fluorescence, 925–68071, Li-Cor Biosciences, Cambridge, UK) was used to detect actin (the loading control).

The LI-COR Odyssey CLx imaging system was used to image the gels (LI-COR Biosciences UK Ltd, Cambridge, UK). Images were analyzed using Image Studio Lite (LI-COR Biosciences UK Ltd, Cambridge, UK). The normalization calculation was as follows: lane normalization factor (LNF) = actin signal from lane/highest actin signal in blot and the normalized signal = (3-nitrotyrosine signal/LNF) x 10^−4^. Western blots of peripheral blood erythrocytes were performed as described above but using 8–16 % pre-cast gradient gels (Bio-Rad) and a Bio Rad Trans-Blot Turbo transfer system. The bicinchoninic acid (BCA) assay (Thermo Fisher, Swindon, UK) was used to determine the protein concentration of samples using BSA (0.06–2 mg/ml) as standards. Then, for electrophoresis, 30 μg sample protein was loaded/well. A rabbit polyclonal anti-human hemoglobin antibody (Ab 191183, Abcam, Cambridge, UK) was used to test for the co-localization on western blots of hemoglobin and the 15 kDa nitrated protein; the visualization was performed using an anti-rabbit secondary antibody (IRDye680RD, red fluorescence, product number 925–68071, Li-Cor Biosciences, Cambridge, UK). Human immunoglobulin G was used to assess non-specific binding of secondary antibodies to IgG present within samples (product number 56834, Merck, Gillingham, UK). Additionally, some western blots were stained using Revert total protein stain (LiCor) prior to blocking and antibody incubation. One of the most abundant proteins in human erythrocytes is hemoglobin. The equal protein loading of the erythrocyte lysate samples when they were applied to the wells of the polyacrylamide gels prior to electrophoresis, was established by determining the sample hemoglobin concentrations using Drabkin’s assay [52]. Drabkin’s reagent (catalog number D5941) was purchased from Sigma. The assay was conducted according to the manufacturer’s instructions, using a standard curve from dilutions of cyanomethemoglobin (180 mg/ml) and reading the absorbance at 540 nm. Based on Drabkin’s assay, 30 μg of hemoglobin was loaded into each well of the gel.

### 2.5 Analysis of nitrated human brain proteins by mass spectrometry

Proteins separated by reducing SDS-PAGE (12 % acrylamide gel), were excised from the Coomassie-stained gel, and trypsin-digested and prepared for mass spectrometry as previously described [53]. Samples were analyzed by liquid chromatography tandem mass spectrometry (LC-MS/MS) using an Ultimate 3000 high pressure liquid chromatography (HPLC) system (Dionex, UK). The peptides were separated on a nano-HPLC column (0.075 × 150 mm, 3 μm, PepMap C18; Thermo Scientific, Hemel Hempstead, UK) at a flow rate of 300 nL/min using a gradient elution running from 2 % to 45 % aqueous acetonitrile (0.1 % formic acid) over 60 min. The HPLC was coupled to a 5600 TripleTOF mass spectrometer (Sciex, Warrington UK). Ionization of the peptides was achieved with the following settings: spray voltage 2.4 kV, source temperature 150 °C, declustering potential 50 V and a curtain gas of 15 V. High resolution TOF MS mode was used to collect parent ion scans in positive mode, from 400 to 1200 Da for 200 ms. MS/MS data were collected using information-dependent acquisition selecting the 10 most intense ions with the following criteria: +2 to +5 charge states and a minimum intensity of 500 counts-per-second, using dynamic exclusion for 20s, 250 ms acquisition time, and standard settings for rolling collision energy settings. The generated data were then analyzed using the Mascot statistical software v 2.3.2 (Matrix Science, London, UK [54]. Scores >50 indicated identity or extensive homology at the p = 0.05 probability cut off [54]. Mascot Daemon (MatrixScience, 2014) was also used to search Swiss-Prot for protein identifications [55].

### 2.6 Electrochemiluminescence-linked immunosorbent assay for total protein 3-nitrotyrosine in human brain tissue

The total protein-bound 3-nitrotyrosine content of the tissue was measured by an electrochemiluminescence-based immunosorbent assay (ECLISA), as described previously [56]. The anti-nitrotyrosine antibody (1 μg/ml) (Cambridge Biosciences, Cambridge, UK) was added to a “Standard Bind” single spot 96-well plate (MSD, Maryland, USA) and incubated at 4 °C overnight. The plate was blocked with blocker A (product number R93BA-4) and standard/samples (25 μl) added. After washing (0.05 % Tween-20 in PBS), a biotinylated version of the same anti-nitrotyrosine antibody (2 μg/ml) was used with SULFO-TAG labelled streptavidin (1:500) coupled to a ruthenium complex. Read buffer (50 % Read Buffer T 4x, 50 % H_2_O) was placed into each well and the plate immediately read on an Electrochemiluminesence Sector Imager 2400 (Meso Scale Discovery (MSD), Maryland, USA). A nitrated BSA standard (0.04 nM–10 nM) dissolved in a dilution buffer (1 % blocker A in PBS) or 1 % blocker A in PBS used as a blank. The standard curve was run with 0.1 % lysis buffer, equivalent to that present in the samples. Samples were measured in duplicate. The intra-assay CV for brain tissue (n = 8) was 6.4 %.

### 2.7 Nitrate and nitrite analyses of human brain tissue by ozone-based chemiluminescence

Samples (human brain frontal lobe homogenates prepared according to the method described under Section 2.1) were deproteinized using a modification of the zinc sulfate precipitation technique [57], using 0.5 M NaOH and 10 % ZnSO_4_. Samples were vortexed and incubated at room temperature for 15 min, then centrifuged at 17,500 *g* for 5 min. The supernatant was analyzed for NO_3_^−^ and NO_2_^−^ concentrations using a nitric oxide analyzer (Sievers NOA 280; Analytix Ltd, Durham, UK) [58]. Samples were refluxed in 0.1 M vanadium chloride (VCl_3_) in 1 M HCl at 95^o^C (for NO_3_^−^) or sodium iodide and glacial acetic acid at 35 °C (for NO_2_^−^). The NO produced then reacts with ozone to produce a luminescence signal. Standard curves were constructed using known concentrations of sodium nitrate or sodium nitrite. Samples were analyzed in duplicate. The assays’ between-batch coefficients of variation were 6.1 % for NO_2_^−^ and 5.8 % for NO_3_^−^.

### 2.8 Statistical analysis

A test of normality (Shapiro-Wilks) showed that the data values had a skewed distribution and did not satisfy normality. Data were therefore analyzed using non-parametric tests; one-way ANOVA (Kruskal-Wallis), students *t*-test (Mann Whitney U) and Spearman’s rank correlation, r_s_). A Bonferroni post-test was applied to cases of multiple correlations against the same data set. A P value of less than 0.05 was considered statistically significant.

## 3 Results

### 3.1 Identity of 3-nitrotyrosine-containing proteins in human frontal lobe brain tissue

**Fig. 1:**
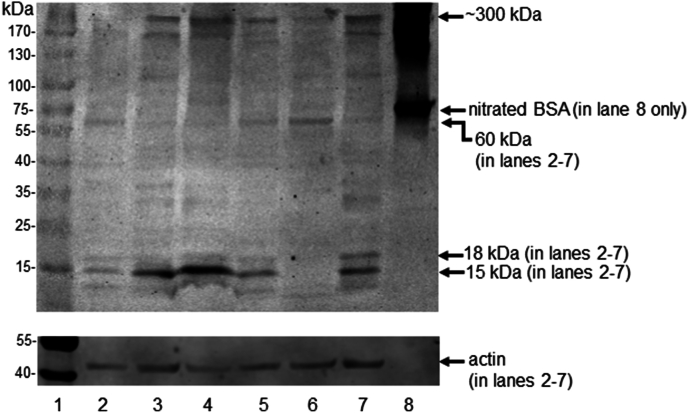
Western blot analysis of representative 3-nitrotyrosine-positive bands in the frontal lobe brain tissue homogenates from patients with Alzheimer’s disease, vascular dementia or non-dementia controls. The upper blot shows 3-nitrotyrosine-positive bands at 300, 60, 18 and 15 kDa. The 15 kDa band was found to be nitrated in nearly all patient samples. Lane 1, protein molecular weight markers; lanes 2 and 3, brain tissue extracts from Alzheimer’s disease (AD) patients; lanes 4 and 5, brain tissue extracts from vascular dementia (VaD) patients; lanes 6 and 7, brain tissue extracts from non-dementia (ND) control individuals; with 60 μg of protein loaded/well. Lane 8, nitrated BSA (1 μg/well; prepared in the laboratory according to the procedure described in section 2.3) was used as a positive control for the presence of 3-nitrotyrosine within a polypeptide. The blot shown (which has been rendered as a grayscale image) was obtained by the immunoblotting of a 12 % cross-linked polyacrylamide gel after reducing SDS PAGE. The lower blot shows the corresponding immunoblot of cytoskeletal β-actin, which was used as a loading control (bands indicated with arrow). This figure shows a representative immunoblot selected from a total of 10 immunoblots performed, in which each of the blots contained samples from different patients.

Human brain homogenates from AD, VaD patients and ND controls (see Table 1 for patient and control demographics) were analyzed by reducing SDS-PAGE (with 12 % gels) followed by western blotting. Western blotting for 3-nitrotyrosine-containing proteins was performed to determine: (1) whether 3-nitrotyrosine-containing proteins were detectable; (2) if so, the number of 3-nitrotyrosine-containing proteins, their molecular weight distribution and relative staining intensities; and (3) if there was a different pattern of nitration between patient groups. Several nitrated protein bands between 10 and 1000 kDa were observed in western blots of frontal lobe brain tissue samples (from both dementia and ND controls). The most prominent nitrated protein bands were seen at approximately 300, 60, 18 and 15 kDa and a representative western blot is shown in Fig. 1.

The 300, 60, 18 and 15 kDa nitrated protein bands (Supplementary Fig. 1) were excised from a Coomassie-stained gel, and peptides present in the bands were identified by mass spectrometry. The data for the proteins with the top ten protein abundance index (emPAI) scores for each of the excised bands are shown in Supplementary Table 1, Parts A-G. Mass spectrometry analysis identified the nitrated protein band with a molecular weight of approximately 300 kDa as non-erythrocytic spectrin-α and spectrin-β (Supplementary Table 1, Parts A-C). The two major proteins in the 60 kDa band were pyruvate kinase and dihydropyrimidinase-related protein 2 (Supplementary Table 1, Part D). The band excised from the Coomassie gel at 18 kDa, was identified by mass spectrometry as peptidyl-prolyl *cis-trans* isomerase A (Supplementary Table 1, Part E).

In addition to the nitrated protein bands at molecular weights ≥18 kDa, the single protein band at approximately 15 kDa on 12 % crosslinked polyacrylamide gels frequently showed evidence of nitration in human brain tissue, when immunoblotted using an anti-nitrotyrosine antibody, in the AD, VaD and ND groups (Fig. 1). Analysis of this 15 kDa band by LC-MS/MS (Supplementary Table 1, Parts F and G) revealed it to be the α- and β-subunits of hemoglobin, which had not been resolved on the gel due to the close similarity of the molecular weights of the α- and β-subunits (these molecular weights being 15,248 and 15,988 Da, respectively). This 15 kDa band (marked “F” and “G” on the gel, Supplementary Fig. 1) was assigned using the following criteria: (1) the excised protein band was at a migration distance which was consistent with the molecular weights of the α- and β-subunits of hemoglobin; (2) the mass spectrometry-derived ion score was high in relation to the other proteins identified in the band. The two protein subunits, hemoglobin-α and -β, fulfilled both these criteria and had the highest confidence score of 3 (Supplementary Table 1), with a sequence coverage of 69 % and 75 % for the α and β subunits, respectively. Hemoglobin subunits were also identified in other higher molecular weight bands (60 kDa and approximately 300 kDa), although the ion scores for hemoglobin subunits in these bands were not as high as the score seen for the 15 kDa band. This raises the possibility that aggregation of hemoglobin subunits had occurred, possibly as a result of oxidative damage to hemoglobin [59]. These nitrated bands, at 60 kDa and approximately 300 kDa, did not show statistically significant differences in signal intensity between the groups.

**Fig. 2:**
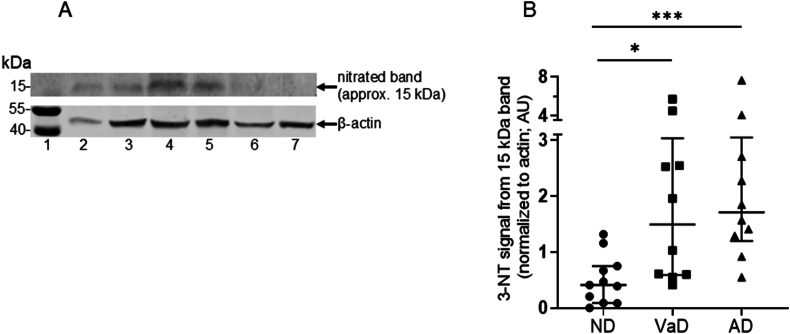
Western blot analysis of the 15 kDa 3-nitrotyrosine-positive band in the frontal lobe brain tissue homogenates from patients with Alzheimer’s disease or vascular dementia, and from non-dementia controls. Panel A shows a representative image of the region of the blot which corresponds to about 15 kDa (upper image) and an image of the region of the same blot which corresponds to protein molecular weights of 40–55 kDa (lower image). The upper image in Panel A shows an immunoblot obtained when probing with the anti-nitrotyrosine antibody and the lower image shows the immunoblot obtained when the same gel was probed with the antibody to cytoskeletal β-actin. The latter was used as a loading control (bands indicated with arrow). Lane 1, protein molecular weight markers (2 μl/well, according to the manufacturer’s instructions); lanes 2 and 3, brain tissue extracts from Alzheimer’s disease (AD) patients; lanes 4 and 5, brain tissue extracts from vascular dementia (VaD) patients; lanes 6 and 7, brain tissue extracts from non-dementia (ND) control individuals; from different patients to those shown in Fig. 1. There was 30 μg of protein loaded/well in lanes 2–7. The blot shown was obtained by the immunoblotting of a 12 % cross-linked polyacrylamide gel after reducing SDS PAGE. Panel B shows the fluorescence intensity of the 15 kDa 3-nitrotyrosine band in all of samples analyzed within the control group and the two patient groups. The graph shows the median and IQR values for each group, as long and short horizontal bars, respectively. Image analysis of the immunoblots was performed, to obtain a signal intensity for each 15 kDa band, which was normalized using the actin signal to determine a lane normalization factor. The normalized median 3-nitrotyrosine-associated fluorescence intensities (IQR) for the 15 kDa band were: ND, 0.41 (0.09–0.75) AU, n = 7; VaD, 1.50 (0.59–3.04) AU, n = 9; and AD, 1.71 (1.20–3.05) AU, n = 9. The fluorescence intensity was significantly higher in both the AD and VaD groups compared to ND control group (Kruskal-Wallis test, P < 0.0005, followed by Mann Whitney *U* test, P < 0.05).

An immunoblot for the nitrated 15 kDa band (along with β-actin bands) present in homogenized human brain tissue from AD, VaD and ND patients is shown in Fig. 2 (Panel A). Semi-quantitative analysis of the nitrated hemoglobin band present in homogenized human brain tissue from AD, VaD and ND patients was performed, assessing the nitrated hemoglobin band fluorescence when normalized to the staining intensity of the housekeeping protein, β-actin, using LiCor software. Fig. 2 (Panel B) shows that the median normalized fluorescence intensities of nitrated hemoglobin were: ND, 0.41 (0.09–0.75) AU; VaD: 1.50 (0.59–3.04) AU; and AD: 1.71 (1.20–3.05) AU. Thus, hemoglobin nitration was detected in all groups (including ND controls). However, the median normalized fluorescence intensities for nitrated hemoglobin were significantly different between groups (Kruskal-Wallis test; P < 0.005) and were observed to be higher in both the AD and VaD groups (n = 9 in each group) compared to the ND control group (n = 8; Mann Whitney *U* test for AD *versus* ND: P < 0.005 and for VaD *versus* ND: P < 0.05).

### 3.2 Comparison of Braak stages with hemoglobin nitration levels

**Fig. 3:**
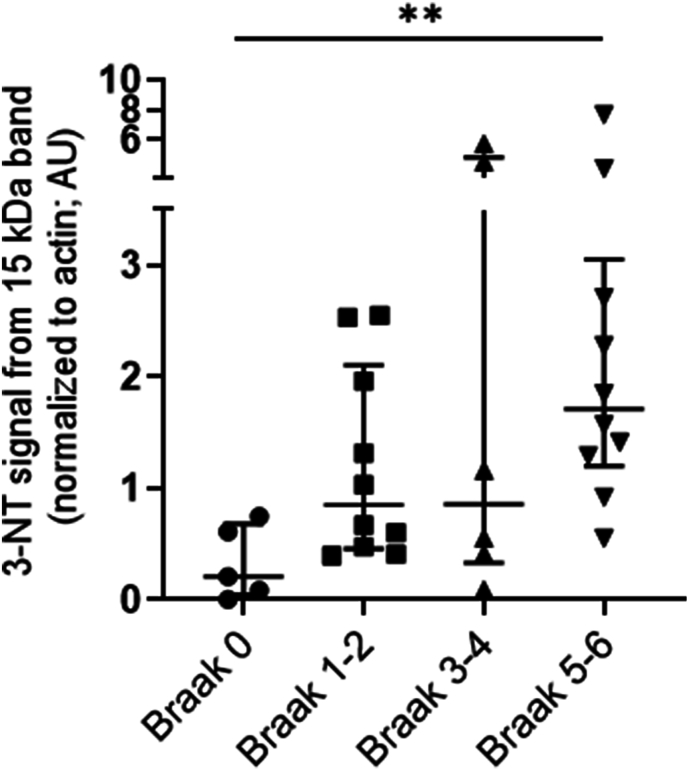
Normalized intensities of the 3-nitrotyrosine staining of the 15 kDa band in Alzheimer’s disease, vascular dementia and non-dementia groups according to Braak classification. The normalized 3-nitrotyrosine staining intensity of the 15 kDa band in western blots of brain homogenates was grouped according to Braak stage. The graph shows the median and IQR values for each group, as long and short horizontal bars, respectively. There were higher amounts of nitrated 15 kDa protein bands in the Braak 5–6 stage group (n = 10) than in the Braak 0 stage group (n = 5); Mann Whitney *U* test, ∗∗P < 0.005. The median 3-nitrotyrosine-associated fluorescence intensities (IQR in brackets) were: Braak stage 0, 0.20 (0.04–0.68); Braak stages 1–2, 0.85 (0.46–2.10); Braak stages 3–4, 0.86 (0.33–4.80); and Braak stages 5–6, 1.71 (1.20–3.05) AU. 3-Nitrotyrosine, 3-NT.

Braak stages were employed to describe the extent of the pathology-based evidence for the involvement of brain regions in disease-associated changes. The normalized median 15 kDa band (nitrated hemoglobin) band intensities in the patient groups at various Braak stages (Fig. 3) were: stage 0, 0.20 (0.04–0.68) AU; stages 1–2, 0.85 (0.46–2.10) AU; stages 3–4, 0.86 (0.33–4.80) AU; stages 5–6, 1.71 (1.20–3.05) AU. The median nitrated hemoglobin was different between groups (Kruskal–Wallis test, P < 0.05) and was found to be increased in individuals with Braak stages 5–6 (n = 10) compared to the group of individuals at Braak stage 0 (n = 5); Mann-Whitney *U* test, P < 0.005.

### 3.3 Western blotting of 3-nitrotyrosine-containing proteins in peripheral blood erythrocytes

**Fig. 4:**
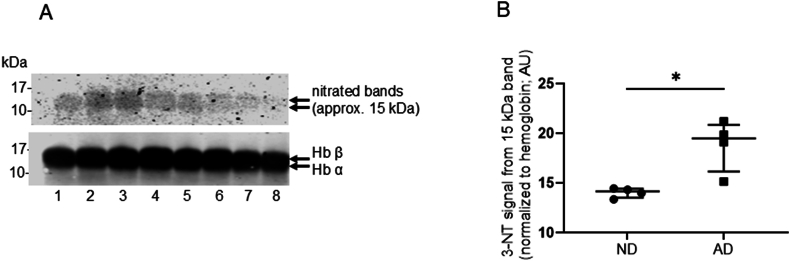
Western blot results showing hemoglobin nitration in erythrocytes from Alzheimer’s disease patients, compared to non-dementia controls. Panel A shows a representative blot probed with (upper part) the anti-nitrotyrosine antibody and (lower part) the anti-hemoglobin antibody. A commercial anti-hemoglobin antibody was observed to give a fluorescence signal from two closely migrating bands (almost indistinguishable on the blot shown) at about 15 kDa, using an 8–16 % gradient gel. Lanes 1–4, peripheral blood erythrocyte lysates from Alzheimer’s disease (AD) patients and lanes 5–8 peripheral blood erythrocyte lysates from non-dementia (ND) controls. Hemoglobin was measured using Drabkin’s assay, and 30 μg of hemoglobin was loaded onto each well. The proteins were transferred onto nitrocellulose membranes, then probed with both anti-hemoglobin and anti-nitrotyrosine antibodies. Panel B shows the results of semi quantitative image analysis when using a lane normalization factor obtained from the hemoglobin signal. The graph shows the median and IQR values for each group, as long and short horizontal bars, respectively. These results show that the extent of nitration of hemoglobin was significantly increased in AD patients (n = 4) compared to ND controls (n = 4) (Mann-Whitney *U* test ND vs AD, ∗P < 0.05). 3-Nitrotyrosine, 3-NT; hemoglobin β-subunit, Hb β; hemoglobin α-subunit, Hb α.

Human erythrocyte lysates were analyzed by SDS-PAGE (gradient 8–16 % gels) followed by western blotting. The blot in Fig. 4 (Panel A) shows the nitrated hemoglobin band in extracts of peripheral blood erythrocyte lysates stained using both anti-nitrotyrosine (upper blot) and anti-hemoglobin antibodies (lower blot). The bands for native hemoglobin and nitrated hemoglobin had indistinguishable electrophoretic mobilities. Image analysis showed anti-3-nitrotyrosine (green) and anti-hemoglobin (red) bands colocalized to give yellow bands (colored image not shown here) giving further evidence that hemoglobin was the 15 kDa protein that was nitrated in erythrocytes. In contrast to the single band for hemoglobin seen on 12 % gels, on an 8–16 % gel, it was possible to resolve the two closely migrating bands corresponding to the α- and β-subunits of hemoglobin (Fig. 4 and Supplementary Fig. 2). Semi-quantitative analysis was performed for the nitrated hemoglobin band fluorescence intensity, using LiCor software. Fig. 4 (Panel B) shows the normalized fluorescence intensity of nitrated hemoglobin in peripheral blood erythrocytes from AD and ND patients. The median fluorescence intensity of nitrated hemoglobin, normalized to hemoglobin, in AD patients was 19.5 (16.2–20.9) AU, which was significantly higher than the nitrated hemoglobin level in healthy control subjects (14.2 (13.5–4.4) AU; Mann Whitney *U* test, P < 0.05, n = 4.

An anti-hemoglobin antibody had been used to probe the blots from electrophoresed samples of human peripheral blood erythrocyte lysates. Therefore, to test whether the 15 kDa band in brain tissue could be immunochemically identified as hemoglobin, reducing SDS PAGE and western blotting experiments were performed using the samples of human brain tissue homogenates, in which the blots were probed with the anti-hemoglobin antibody (as opposed to the anti-actin antibody used in earlier brain tissue analyses) in combination with probing by the anti-nitrotyrosine antibody. Western blots, showing the full-length lanes of the separated proteins from brain tissue homogenates, are presented in Supplementary Fig. 2. It was observed that the nitrated protein bands (green fluorescence) colocalized (yellow) with the hemoglobin bands (red fluorescence) in brain tissue from AD, VaD patients and ND controls.

### 3.4 Western blotting control experiments

In addition to a 3-nitrotyrosine-containing band corresponding to the hemoglobin subunits at 15 kDa, there were also hemoglobin bands detected at 30 and 55 kDa (Supplementary Fig. 2). These higher molecular weight bands may be covalently linked dimers and tetramers of hemoglobin subunits, in which the inter-molecular covalent bonds were stable to reducing SDS PAGE, *i.e.*, the covalent bonds did not correspond to disulfide bridges. However, further experiments were required before being able to rule out the possibility that the higher molecular weight bands might be explained by non-specific binding of the secondary antibodies to human IgG fragments. IgG fragments have similar molecular weights to those predicted for covalently linked dimers/tetramers of hemoglobin subunits (the IgG light chain has a molecular weight of about 25 kDa and the IgG heavy chain has a molecular weight of about 50 kDa). To test whether the bands at 30 and 55 kDa were truly nitrated proteins (and not due to antibody cross-reactivity with IgG chains), commercial purified human IgG, human hemoglobin and nitrated human hemoglobin (synthesized as described in section 2.3) were blotted and probed with primary and secondary antibodies, or with a secondary antibody without the presence of the primary antibody. The results (Supplementary Fig. 3, Panel A) demonstrated that the secondary antibodies showed no significant non-specifical binding to IgG and confirmed that hemoglobin multimers were present in brain samples. A prominent nitrated hemoglobin band at 30 kDa was seen in nitrated hemoglobin synthesized in the laboratory (Supplementary Fig. 3, Panel A, lane 3), which was also seen in patient samples (Supplementary Fig. 2). A similar dimeric band was observed by Denicola et al. [60] after both purified human Hb and erythrocytes were exposed to ONOO^−^. Supplementary Fig. 3 (Panel A) confirmed there was very low non-specific binding of the secondary antibodies to hemoglobin at similar fluorescence intensities (600 AU) as seen in sample blots. It was noted that there was an absence of non-specific binding of the secondary antibodies to nitrated proteins on the blot. Next, as hemoglobin displays autofluorescence [61], western blotting was employed to determine if hemoglobin displayed an inherent fluorescence, without being incubated with primary or secondary antibodies and Supplementary Fig. 3 (Panel B) confirmed that hemoglobin fluorescence in the infra-red region used here was minimal. Supplementary Fig. 3 (Panel C) shows that the molecular weight markers imaged in the red channel, 700 nm, and in the green channel, 800 nm, were only visible in the red channel. The markers only became visible in the green channel after the anti-nitrotyrosine antibody was used, confirming that the marker proteins were nitrated and there was no spectral overlap between the red and green channels using the LiCor system. Non-specific binding of secondary antibodies to hemoglobin was assessed by incubating blots with secondary antibodies (without the primary antibodies). In Supplementary Fig. 3 (Panel D) both the anti-β-actin and the anti-3-nitrotyrosine bands displayed a linear increase in fluorescence intensity as the protein load was increased in a sample.

### 3.5 Quantitation by ECLISA of total protein 3-nitrotyrosine levels in brain tissue

Total protein 3-nitrotyrosine levels, adjusted for sample protein concentration, were measured by ECLISA and the median and IQR determined for each group (Supplementary Fig. 4). Total protein 3-nitrotyrosine levels showed a high inter individual variability and there were no statistically significant differences observed between the median values of total 3-nitrotyrosine content: median (IQR) AD, 0.29 (0.19–0.57) pmol nitrated albumin equivalents/mg protein; VaD, 0.36 (0.18–0.40) pmol/mg; and ND controls 0.3 (0.22–0.55) pmol/mg. There was no association between total protein 3-nitrotyrosine levels and patient age, tissue pH or post-mortem interval.

### 3.6 Measurement of nitrite and nitrate levels in brain tissue by ozone-based chemiluminescence

**Fig. 5:**
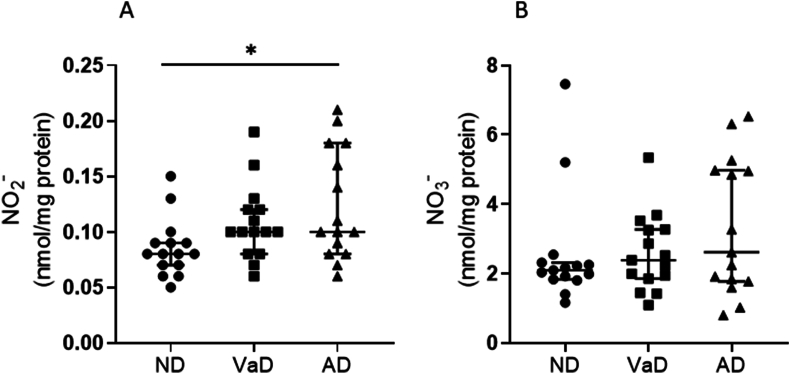
Nitrite and nitrate levels in human brain tissue from Alzheimer’s disease, vascular dementia patients and non-dementia control samples. NO_2_^−^/NO_3_^−^ concentrations were measured by ozone-based chemiluminescence and expressed relative to the amount of protein. Long horizontal lines indicate median values, and short horizontal lines represent IQRs (n = 15 in each group). Panel A shows the levels of NO_2_^−^ in the frontal lobe brain tissue samples from Alzheimer’s disease (AD) vascular dementia (VaD) patients and non-dementia (ND) healthy controls. There was a statistically significant difference in median NO_2_^−^ levels between the ND and AD groups, (Mann Whitney *U* test, ∗P < 0.05, n = 15). Panel B shows the levels of NO_3_^−^ in the frontal lobe brain tissue from the three groups. There were no statistically significant differences between the median NO_3_^−^ levels in the different groups.

The median NO_2_^−^ levels were: ND, 0.08 (0.07–0.09); VaD, 0.11 (0.08–0.12); and AD, 0.12 (0.08–0.18) nmol/mg protein. The median NO_2_^−^ level was significantly different between lysates of frontal lobe brain tissue (Kruskal–Wallis test, P < 0.05, n = 15 in each group). The median NO_2_^−^ level (Fig. 5, Panel A) was significantly higher in lysates of frontal lobe brain tissue from AD participants than in ND controls (Mann Whitney *U* test, P < 0.05). There was no significant difference in median NO_2_^−^ levels between VaD and either of the other groups. There were no statistically significant differences between the three groups when considering median NO_3_^−^ concentrations (Fig. 5, Panel B): ND, 2.1 (1.8–2.3) nmol/mg protein; VaD 2.4 (1.9–3.3) nmol/mg protein; and AD 2.6 (1.8–4.9) nmol/mg protein**.** Although equivalent areas of brain tissue were excised from donated brains, the median protein concentration (IQR) was lower in the lysates from the AD group (10.1 (6.1–12.3) mg/ml) than in the lysates from either the VaD group (12.8 (10.6–15.3) mg/ml) or the ND individuals (13.4 (9.7–18.5) mg/ml); P < 0.05 and P < 0.005, respectively. Thus, NO_3_^−^ and NO_2_^−^ levels were expressed as nmol/mg of protein to account for the differences in sample protein concentrations. There were no statistically significant correlations between either NO_2_^−^ or NO_3_^−^ levels and patient age, sex, tissue pH or post-mortem interval. Brain NO_3_^−^ and NO_2_^−^ levels were positively correlated with each other (Spearman’s rank correlation, r_s_ = 0.53, P < 0.0001). Brain NO_3_^−^ levels were found to correlate positively with 3-nitrotyrosine (r_s_ = 0.42, P < 0.05). NO_2_^−^ levels did not show a statistically significant correlation with 3-nitrotyrosine. There was a trend towards an inverse association between brain NO_2_^−^ and amyloid-β deposition (Spearman r = −0.59, P = 0.06, n = 11), although the sample size was small.

### 3.7 *Analysis of sex-dependent differences between disease and control groups in relation to the levels of hemoglobin nitration, total protein 3-nitrotyrosine, NO*_*2*_^*−*^*, and NO*_*3*_^*−*^*in brain tissue*

Western blotting analysis showed that the median fluorescence intensity of the nitrated hemoglobin band (normalized to actin) was significantly higher in both the AD and VaD groups compared to ND controls when male and female participants were analyzed together (Section 3.1). When male and female participants were analyzed separately, there were clear differences in brain levels of hemoglobin nitration, total 3-nitrotyrosine, NO_2_^−^, and NO_3_^−^ levels according to sex (Supplementary Fig. 5)**.** Only females showed significantly higher (P < 0.05) normalized hemoglobin nitration levels in AD samples (1.85 (1.11–5.88) AU, n = 5) compared with ND people (0.67 (0.39–0.75) AU, n = 3). In males alone there were no statistically significant differences between the median normalized hemoglobin nitration levels between groups, AD: 1.85 (0.77–2.60) AU, n = 4; VaD: 0.61 (0.51–5.11) AU, n = 5; and ND: 0.79 (0.17–1.28) AU, n = 4. The median NO_2_^−^ levels were significantly higher in homogenates of frontal lobe brain tissue from all the AD participants (male plus female) compared to all ND controls (Section 3.4). However, in female participants alone the median (IQR) brain tissue NO_2_^−^ level was significantly higher in both the AD and VaD groups compared to ND controls, the values being AD: 0.11 (0.10–0.19) nmol/mg protein, n = 10; VaD: 0.10 (0.10–0.13) nmol/mg protein, n = 7; and ND: 0.09 (0.07–0.09) nmol/mg protein, n = 6 (AD v ND, P < 0.05; VaD v ND, P < 0.05). For male participants alone, the median (IQR) brain tissue NO_2_^−^ levels did not show any statistically significant differences between groups: AD: 0.08 (0.08–0.16) nmol/mg protein, n = 5; VaD: 0.10 (0.07–0.12) nmol/mg protein, n = 8; and ND: 0.08 (0.07–0.11) nmol/mg protein, n = 9 (Supplementary Fig. 5).

## 4 Discussion

### 4.1 Hemoglobin nitration in frontal lobe brain tissue samples

We observed 3-nitrotyrosine-containing protein bands on western blots at 300, 60 and 18 kDa which were subsequently assigned to specific proteins by mass spectrometry analysis. The two major proteins in the band excised from the Coomassie gel at 60 kDa, were identified as pyruvate kinase and dihydropyrimidinase-related protein 2. These nitrated proteins were previously identified in AD brain samples by Butterfield et al. [62]. Also in agreement with the observations of Butterfield et al., the 18 kDa band was identified by mass spectrometry as peptidyl-prolyl *cis-trans* isomerase A (Supplementary Table 1) [39,42,62]. In addition to these nitrated proteins, we observed a dominant nitrated band at 15 kDa, and mass spectrometry analysis identified this band as hemoglobin. Mass spectrometry data from the excised 15 kDa bands were assessed according to: (a) whether the molecular weight of the identified polypeptide was consistent with the relevant band’s electrophoretic mobility, and (b) whether the ion score was high in relation to the other proteins identified in the band. We also went on to consider whether (c) the identified protein was already known to be a protein which is present in adult brain tissue (*vide infra*). The two protein subunits, hemoglobin-α and -β, fulfilled all these criteria and had the highest confidence score of 3. This does not completely exclude the possibility that the 15 kDa band may have contained a relatively low abundance protein, which was highly nitrated. However, our data strongly support the assignment of the nitrated 15 kDa band to hemoglobin based on the comparative ion and emPAI scores, which were markedly higher than any other identified protein. 10.13039/100014337Furthermore, in support of assigning the nitrated band to hemoglobin, a commercial anti-hemoglobin antibody was observed to co-localize with this nitrated 15 kDa band in western blots of brain tissue homogenates. The detection of hemoglobin (an erythrocytic protein) and, moreover, the presence of the nitrated form of hemoglobin, in human frontal lobe brain samples was surprising. To our knowledge, the extent of hemoglobin nitration in AD brain tissue has not been reported previously.

A possible explanation for the absence of any reports of hemoglobin nitration, in earlier immunoblotting studies of brain tissue extracts from AD patients, may be the use of chemiluminescence-based detection techniques for the development of blots. Chemiluminescence-based detection methods for immunoblotting often use a horseradish peroxidase-labelled secondary antibody, whereby the detected luminescence of antibody-stained bands is triggered by the addition of luminol and hydrogen peroxide. Horseradish peroxidase catalyzes the oxidation of luminol by hydrogen peroxide. However, hemoglobin itself displays a pseudo-peroxidase activity such that it acts in the same way as horseradish peroxidase, to produce a luminescence signal even in the absence of any binding of the peroxidase-labelled antibody [63]. Thus, a positively stained band corresponding to hemoglobin could have been dismissed as an artefact due to this pseudo-peroxidase activity. Conceivably, it is the advent of fluorescence-labelled antibodies, such as were used in the present study, that has facilitated the identification of nitrated hemoglobin.

The presence of hemoglobin in the brain samples used in this study may be explained by the samples being small blocks of post-mortem frozen brain tissue, which were then homogenized and analyzed. These tissue samples will have contained blood vessels which will, in turn, have trapped erythrocytes with high cellular levels of hemoglobin. Another possibility is that the detected hemoglobin was produced by neurons, as rat and human neurons have been shown to express hemoglobin [64]. It remains unclear whether the hemoglobin detected in the analyzed brain tissue samples was derived from erythrocytes or neurons. Human and rat neuronal cells also contain neuroglobin, a protein with a relatively close sequence homology to hemoglobin [65]. However, analysis of the peptide sequences identified by mass spectrometry, in the present study, confirmed that the protein was hemoglobin rather than neuroglobin.

In a limited number of patients, we also found that peripheral blood erythrocyte lysates from AD patients and ND control subjects contained a dominant nitrated 15 kDa band, which stained with an anti-hemoglobin antibody on western blots. Based on the analysis of western blots, the extent of hemoglobin nitration in brain samples (expressed relative to hemoglobin band intensity, Supplementary Fig. 2) was several-fold higher than in the erythrocytes (Fig. 4). However, this comparison between brain and erythrocyte samples was not quantitative, as it is unlikely that there is preservation of the linearity of the concentration-response curve for the anti-hemoglobin fluorescence response across the entire broad range of hemoglobin concentrations in brain tissue and erythrocytes (relatively low concentrations of hemoglobin in brain tissue, to the much higher concentrations in erythrocytes). Nevertheless, the clear increase in the proportion of nitrated hemoglobin in brain tissue, compared to erythrocytes, is consistent with nitration originating in the brain. Some of the observed hemoglobin nitration may have been extravascular, following the leakage of hemoglobin into the parenchyma. Blood components may enter the brain tissue during micro-bleeds [66] or with increased vascular permeability [67]. The presence of elevated hemoglobin polypeptides in the AD cerebellum was reported as being a likely consequence of blood vessel leakage [68]. Neuroinflammation and oxidative stress in AD are associated with extracellular iron and hemoglobin accumulation in grey matter brain regions [69]. ONOO^−^-mediated nitrative damage to hemoglobin may be associated with the release of heme from hemoglobin [70]. Cellular heme is a substrate for the enzyme heme oxygenase, which (i) catalyzes the degradation of heme to free iron, biliverdin, and carbon monoxide [23] and (ii) has been implicated in the pathogenesis of AD [71]. Free iron has been implicated in oxidative damage as it a powerful catalyst of free radical reactions [72].

Brain tissue homogenates were loaded onto the gels with equal amounts of protein/well, as determined by the BCA assay. However, variable staining intensity was observed for the actin band between samples (Fig. 1, Fig. 2). β-Actin is part of the cell cytoskeleton and the proportion of protein from cells (*versus* protein from the extracellular matrix) may vary in brain tissue samples, depending on the cellular density of each brain tissue biopsy. Western blotting studies by other researchers showed granzyme B-induced cleavage of β-actin in brains of rats following 10-min and 2-h focal ischemia [73]. In relation to the collection of post-mortem human brain tissues used in the present study, it is possible that the duration of ischemic episodes may have varied, leading to varying amounts of intact β-actin being present.

Therefore, after it had been discovered during the present study that the nitrated band in brain homogenates was hemoglobin, and that the β-actin varied between samples, normalization in the subsequent part of the study (Fig. 4) was based on the total hemoglobin band as detected by western blotting using an antibody against native hemoglobin. It was observed that the intensity of the hemoglobin band was less variable between patient samples containing the same amounts of loaded total protein, compared with the variability in the actin band. In a subset of 11 brain samples, there were sufficient sample volumes to allow the determination of hemoglobin nitration levels when normalized in two different ways: nitrated hemoglobin was normalized to the anti-actin fluorescence intensity, and nitrated hemoglobin was also normalized to the anti-hemoglobin fluorescence intensity. In this subset, there was a positive correlation between the nitrated hemoglobin levels when calculated using the two different methods for normalization (Spearman’s rank correlation, r_s_ = 0.68, P < 0.05, n = 11). The median values (IQR) for the normalized hemoglobin band intensities, as obtained using the anti-hemoglobin antibody in western blots, were not significantly different when comparing the homogenates from AD patients and ND controls: AD 151.5 (98.3–210.5) AU, n = 8; VaD 171.0 (95.5–289.0) AU, n = 9; and ND 174.0 (139.5–297.0) AU, n = 9. Thus, there was an increased proportion of hemoglobin which was nitrated, rather than there being more hemoglobin present in AD and VaD brain samples compared to ND brain samples.

### 4.2 The extent of hemoglobin nitration is increased in frontal lobe brain tissue from AD and VaD patients compared to non-dementia control patients, but this is not the case for some of the other nitrated proteins in the brain

The normalized levels of the nitrated hemoglobin band were increased in patients with AD or VaD, compared with ND control individuals. Nitrated hemoglobin also showed increased levels in patients with higher Braak stages. Additional nitrated proteins observed in the brain samples analyzed in the present study were peptidyl-prolyl *cis-trans* isomerase (also known as cyclophilin A) and dihydropyrimidinase related protein 2 (Supplementary Table 1). Peptidyl-prolyl *cis-trans* isomerase has been implicated in cerebrovascular and neurodegenerative pathologies [[74], [75], [76]]. Dihydropyrimidinase related protein 2 is involved in axonal growth and guidance and was shown to be significantly increased in AD brain, suggesting a role for the impaired mechanism of neural network formation in AD [77]. In the present study, the brain tissue from AD patients exhibited significantly increased median levels of normalized nitration within the 18 kDa band (peptidyl-prolyl *cis-trans* isomerase) compared to ND, but there was no significant difference between VaD and ND: AD, 0.52 (0.23–3.91); VaD, 0.13 (0.02–2.00); ND, 0.06 (0.04–0.17) AU (Mann-Whitney *U* test, ND v AD, P < 0.05; ND v VaD, P = 0.48, n = 10 in each group). For the 60 kDa band from frontal lobe brain tissue, which contained dihydropyrimidinase related protein 2, there were no statistically significant differences between groups, in terms of the median (IQR) values of normalized nitration: AD, 0.19 (0.03–0.59); VaD, 0.26 (0.06–0.84); ND, 0.13 (0.03–0.24) AU, n = 10 in each group. Similarly, the 300 kDa band, containing non-erythrocytic spectrin-α and -β, did not display any statistically significant differences between the groups, in relation to the median (IQR) values for normalized nitration: AD, 0.62 (0.17–1.62); VaD, 0.26 (0.07–1.00); and ND, 0.11 (0.01–0.67) AU, n = 10 in each group.

### 4.3 The extent of hemoglobin nitration is increased in erythrocytes from AD patients compared to non-dementia controls

The extent of hemoglobin nitration was increased in peripheral blood erythrocyte lysates from a small number of AD patients compared to ND controls. Hemoglobin is a major protein constituent of blood and performs the crucial role of carrying oxygen to tissues. The slow turnover of erythrocyte hemoglobin (half-life of approximately 120 days) may explain why hemoglobin accumulates nitrative damage and acts as a “sink” for chemical nitrating species. Hemoglobin accumulates covalent chemical modifications, as demonstrated by the presence of glycated hemoglobin (hemoglobin A1c; HbA1c) in patients with diabetes. HbA1c is a useful clinical marker of long-term exposure to high glucose levels [78]. Thus, nitrated hemoglobin, present within both the brain and peripheral blood, may constitute a novel marker of long-term exposure to nitrative/oxidative stress in AD patients. Studies with matched samples of both brain tissue and peripheral blood erythrocytes from the same patients would further elucidate the involvement of nitrated hemoglobin in blood and brain tissue.

The clinical diagnosis of AD is reliant on the exclusion of other causes for the decline in cognitive function/dementia and will typically allow a diagnosis of either ‘possible’ or ‘probable’ AD [79]. To date, the most accepted laboratory method for AD diagnosis in biofluids from living patients is the measurement of amyloid-β (1–42), total tau (T-tau), and phospho-tau-181 (P-tau181) in CSF, collected by lumbar punctures [80]. As CSF sampling is an invasive procedure, new biomarkers in more easily accessible bodily fluids, such as whole blood from which erythrocytes can be readily isolated, continue to be sought. Blood–brain barrier damage in AD may promote the movement of proteins between brain and blood [81,82]. It has been shown that amyloid-β and tau levels increase in blood after crossing the blood-brain barrier [83]. Crystals composed of fibrils on erythrocytes were identified as biomarkers of AD pathology [84] along with oxidative stress indicators [85,86].

### 4.4 *Suggested* s*ex-dependent differences between disease and control groups in relation to the levels of hemoglobin nitration, NO*_*2*_^*−*^*, NO*_*3*_^*−*^*and 3-nitrotyrosine in brain tissue*

We observed that when the sample population was stratified in a sex-based analysis, there were sex-dependent differences between AD, VaD and ND groups in the levels of NO_2_^−^ and hemoglobin nitration. Sex specific differences were also found by Sharma et al. [87], where advanced oxidized protein products, such as oxidized albumin, leading to elevated levels of carbonyl groups of albumin, were measured. AD females displayed lower levels of the advanced oxidation end-product, carboxyethyllysine, compared to AD males. Plasma levels of 3-nitrotyrosine were higher in male AD patients compared to healthy controls [87], but no such differences were observed in females. Furthermore, significantly higher concentrations of NO and ONOO^−^ were observed in platelets from male AD patients compared to female AD patients [88]. Sharma et al. [87] discussed the role of anti-oxidative effects of estrogens and menopause, which may play a role in observed sex-specific differences.

### 4.5 Hemoglobin as a target for nitration in the AD brain, and the potential role of hemoglobin nitration in the pathogenesis of AD

Proteins from human AD brain tissue [34] and CSF [36] have been previously observed to contain 3-nitrotyrosine. Based on the observations in the present report, it appears that hemoglobin should be added to the list of proteins which are targeted for nitrative damage in the brain. Protein nitration is a post-translational modification which is increased under nitro-oxidative stress *in vivo*, with important pathophysiological consequences associated with inflammatory, neurodegenerative, and cardiovascular disorders [89]. In the present study, analysis by reducing SDS-PAGE showed covalently linked dimers, and covalently linked tetramers, of hemoglobin. This finding is consistent with an earlier observation that, after hemolysis and SDS-PAGE analysis, free hemoglobin exists in plasma as tetramers and αβ-subunit heterodimers, with a predominant dimer state [90]. Treatment of ultra-purified hemoglobin with H_2_O_2_ generates ferryl hemoglobin leading to the formation of covalently cross-linked α/β-globin dimers and tetramers [91]. Tyrosyl radicals can form covalent dityrosine bonds, leading to inter- and intra-molecular cross-linking and to ferrylhemoglobin polymerization [92]. Under pathophysiologic conditions, oxidized and cross-linked hemoglobin dimers and tetramers were detected in human CSF [93].

Under inflammatory conditions, superoxide reacts rapidly with NO to form ONOO^−^. When hemoglobin is exposed, *in vitro*, to nitrating agents such as ONOO^−^, nitration occurs at Tyr-24 and Tyr-42 in α-globin, and at Tyr-130 in β-globin [94]. *In vivo*, a potential mechanism of 3-nitrotyrosine generation is via the MPO–H_2_O_2_–NO_2_^−^ system. Our results have shown that NO_2_^−^ was increased in the frontal lobe of the AD brain and therefore it appears NO_2_^−^ is available in the brain as a substrate for MPO. Additionally, oxyhemoglobin undergoes a slow auto-oxidation, producing the superoxide anion radical which, in the presence of intracellular superoxide dismutase, yields H_2_O_2_ [95]. In the presence of NO_2_^−^, which we have found here to be elevated in AD brain tissue, and H_2_O_2_, hemoglobin demonstrates pseudo-halide peroxidase activity, resulting in self-nitration [96]. H_2_O_2_ oxidizes ferric (FeIII)-hemoglobin to ferryl (FeIV)-hemoglobin and, in the presence of NO_2_^−^, Fe(IV)-hemoglobin is reduced to Fe(III)-hemoglobin with the simultaneous oxidation of NO_2_^−^ to the nitrating agent, nitrogen dioxide (NO_2_), thereby self-nitrating tyrosine residues within hemoglobin. Hemoglobin nitration has been detected *in vivo* by mass spectrometry in human blood [94] and is increased in cigarette smokers [94] and in type 2 diabetes [97]. However, there have been no previous reports of hemoglobin nitration in the human brain, and no studies of the extent of hemoglobin nitration in relation to AD. The self-nitration of hemoglobin may explain the selectivity that we have observed for the nitration of hemoglobin, in comparison with the level of nitration in other brain tissue proteins.

There is experimental evidence suggesting pathways by which nitration reactions involving hemoglobin might contribute to AD pathogenesis [98]. As mentioned above, extracellular hemoglobin has been detected in the human brain, possibly emanating from hemolysis around cerebral micro blood vessels [99]. Hemoglobin binds to extracellular amyloid-β peptide [100] via heme within hemoglobin [101]. Wu et al. [102] reported that hemoglobin is colocalized with amyloid-β deposits in the AD brain, colocalizes with senile plaques in AD brains and promotes amyloid-β oligomer formation. Kummer et al. [103] reported that within the plaques of an AD mouse model (APP/PS1) the nitration of tyrosine 10 in the amyloid-β peptide caused a marked enhancement of amyloid-β aggregation and plaque formation. When heme binds to amyloid-β, the iron center of the heme-amyloid-β complex offers one electron to O_2_ and the Tyr10 residue of the amyloid-β possibly offers another electron [104], forming H_2_O_2_ and a radical on Tyr10 of the amyloid-β peptide. This tyrosyl radical can then result in amyloid-β aggregation or the nitration of the amyloid-β peptide [105,106]. We suggest that the nitration reactions within hemoglobin-amyloid-β complexes, involve not only the nitration of amyloid-β, but also the self-nitration of hemoglobin. Dey and colleagues have reported that heme-bound amyloid-β peptides have peroxidase activity [[107], [108], [109], [110]]. We suggest that the observed presence of nitrated hemoglobin in AD brain tissue reflects the enhanced peroxidase activity of hemoglobin that is colocalized with heme-bound amyloid-β peptides. Thus, in the presence of H_2_O_2_ and NO_2_^−^, heme-bound amyloid-β peptides may catalyze hemoglobin nitration [111], thereby explaining the presently reported detection of 3-nitrotyrosine in AD brain tissue.

The accumulation of nitrated/oxidized proteins may result from either an increase in protein nitration/oxidation, or a decline in the degradation of post-translationally modified proteins, or from a combination of both changes. The ubiquitin proteasome system is one of the most important mechanisms for degrading proteins [[112], [113], [114]]. Nitration targets proteins for degradation, and 3-nitrotyrosine-containing proteins undergo faster degradation and removal by the proteasome [115]. However, AD patients exhibit decreased proteasomal activity in the hippocampus, a particularly vulnerable area of the brain during the early stages of the disease [116,117]. Furthermore, the proteasome was inhibited by accumulated oxidized or cross-linked protein aggregates, leading to decreased proteasomal activity and progressive diminution of cellular ability to degrade oxidized proteins in AD [118,119]. Therefore, either an increased rate of formation of nitrated hemoglobin, and/or a decreased rate of removal of nitrated hemoglobin, could explain our current observation of an increased steady-state level of hemoglobin nitration in brain tissue from AD patients compared with ND control tissue.

The potential functional consequences of hemoglobin-catalyzed nitration may be relevant to AD pathogenesis through mechanisms which extend beyond just the deposition of amyloid-β plaques. When proteins are modified by post-translational modifications, thereby generating neoepitopes, this can lead to the production of autoantibodies [120] in association with autoimmunity. Autoantibodies (targeting brain proteins such as amyloid-β plaques) were detected in the CSF of AD patients (reviewed in Ref. [121]). It is conceivable that such autoantibodies are targeted to peptide/protein neoepitopes containing 3-nitrotyrosine. However, the nitration of brain hemoglobin may not be specific to AD, as it may occur in other chronic inflammatory diseases. The synthesis of NO in the human brain is catalyzed by the nitric oxide synthase (NOS) isozymes: neuronal NOS (nNOS), inducible NOS (iNOS), and endothelial NOS (eNOS). Subsequent biochemical reactions of NO (see Introduction) may then cause protein nitration. Pharmacological intervention studies in mouse models of AD using selective inhibitors of NOS isozymes [122] that inhibit protein nitration [123]), as well as NADPH oxidase-2 inhibitors [[124], [125], [126]] and MPO inhibitors [127], may aid in defining the extent to which hemoglobin nitration plays a role in the pathogenesis of AD - or whether nitrated hemoglobin is a consequence of the disease process.

### 4.6 Conclusions

Western blotting of frontal lobe brain tissue revealed that specific proteins were prone to nitration. Mass spectrometry analysis showed, surprisingly, that one of these proteins was hemoglobin - despite hemoglobin being classically regarded as an erythrocytic protein. This identity was confirmed by western blotting using a commercial anti-hemoglobin antibody. The present study showed a significant increase in the extent of nitration of hemoglobin in brain tissue from AD and VaD patients compared to ND controls. Furthermore, the extent of hemoglobin nitration was also increased in peripheral blood erythrocyte lysates from a small number of AD patients compared to ND controls. We propose that increased levels of hemoglobin nitration in erythrocytes may be a biomarker of early AD. There are multiple functional consequences of hemoglobin nitration, as discussed above, which may impinge on the pathogenesis of AD.

## CRediT authorship contribution statement

**M.J. Smallwood:** Writing – review & editing, Writing – original draft, Visualization, Validation, Supervision, Project administration, Methodology, Investigation, Formal analysis, Data curation. **M. Abu Alghayth:** Writing – review & editing, Visualization, Validation, Methodology, Investigation, Formal analysis, Data curation. **A.R. Knight:** Writing – review & editing, Writing – original draft, Visualization, Validation, Methodology, Investigation, Formal analysis, Data curation. **K. Tveen-Jensen:** Writing – review & editing, Visualization, Validation, Software, Methodology, Investigation, Formal analysis, Data curation. **A.R. Pitt:** Writing – review & editing, Visualization, Validation, Supervision, Methodology, Investigation, Formal analysis, Data curation. **C.M. Spickett:** Writing – review & editing, Visualization, Validation, Supervision, Resources, Methodology, Investigation, Formal analysis, Data curation. **D. Llewellyn:** Writing – review & editing, Investigation, Funding acquisition, Conceptualization. **G. Pula:** Writing – review & editing, Resources, Project administration, Investigation, Formal analysis, Data curation. **A. Wearn:** Writing – review & editing, Resources, Methodology, Investigation, Data curation. **A. Vanhatalo:** Writing – review & editing, Supervision, Project administration, Investigation, Formal analysis. **A.M. Jones:** Writing – review & editing, Supervision, Project administration, Investigation, Formal analysis. **P. Francis:** Writing – review & editing, Visualization, Investigation, Formal analysis, Data curation, Conceptualization. **E. Coulthard:** Writing – review & editing, Visualization, Resources, Project administration, Investigation, Formal analysis, Data curation. **P.G. Kehoe:** Writing – review & editing, Visualization, Resources, Project administration, Investigation, Funding acquisition, Formal analysis, Data curation, Conceptualization. **P.G. Winyard:** Writing – review & editing, Writing – original draft, Visualization, Validation, Supervision, Resources, Project administration, Methodology, Investigation, Funding acquisition, Formal analysis, Data curation, Conceptualization.

## Funding

This study was funded by the Science and HASS Strategies Project Development Fund of the University of Exeter (awarded to P.G.W, D.L and P.G.K.). The SWDBB is part of the Brains for Dementia Research program, jointly funded by Alzheimer’s Research UK and the Alzheimer’s Society and is supported by 10.13039/100011699BRACE Dementia Research and the 10.13039/501100000265Medical Research Council, UK. Collection of erythrocytes by the 10.13039/501100000883University of Bristol was funded by Alzheimer’s Research UK, 10.13039/100011699BRACE and 10.13039/100004440Wellcome (Grant number 109067/Z/15/AI).

## Declaration of competing interest

The authors declare that they have no known competing financial interests or personal relationships that could have appeared to influence the work reported in this paper.

## Data Availability

Data will be made available on request.

## References

[1] Sosa-Ortiz A.L., Acosta-Castillo I., Prince M.J. (2012).

[2] Zhu C.W., Sano M. (2006). Economic considerations in the management of Alzheimer’s disease. Clin. Interv. Aging.

[3] Skrobot O.A., O'Brien J., Black S., Chen C., DeCarli C., Erkinjuntti T., Ford G.A., Kalaria R.N., Pantoni L., Pasquier F., Roman G.C., Wallin A., Sachdev P., Skoog I., Ben-Shlomo Y., Passmore A.P., Love S., Kehoe P.G. (2017). The vascular impairment of cognition classification consensus study. Alzheimers Dement.

[4] Brown W.R., Thore C.R. (2011). Review: cerebral microvascular pathology in ageing and neurodegeneration. Neuropathol. Appl. Neurobiol..

[5] Kalaria R. (2002). Similarities between Alzheimer's disease and vascular dementia. J. Neurol. Sci..

[6] Iturria-Medina Y., Sotero R.C., Toussaint P.J., Mateos-Perez J.M., Evans A.C. (2016). Early role of vascular dysregulation on late-onset Alzheimer's disease based on multifactorial data-driven analysis. Nat. Commun..

[7] Benigni A., Cassis P., Remuzzi G. (2010). Angiotensin II revisited: new roles in inflammation, immunology and aging. EMBO Mol. Med..

[8] Kehoe P.G. (2018). The coming of age of the angiotensin hypothesis in Alzheimer's disease: progress toward disease prevention and treatment?. J. Alzheim. Dis..

[9] Heneka M.T., Carson M.J., Khoury J.E., Landreth G.E., Brosseron F., Feinstein D.L., Jacobs A.H., Wyss-Coray T., Vitorica J., Ransohoff R.M., Herrup K., Frautschy S.A., Finsen B., Brown G.C., Verkhratsky A., Yamanaka K., Koistinaho J., Latz E., Halle A., Petzold G.C., Town T., Morgan D., Shinohara M.L., Perry V.H., Holmes C., Bazan N.G., Brooks D.J., Hunot S., Joseph B., Deigendesch N., Garaschuk O., Boddeke E., Dinarello C.A., Breitner J.C., Cole G.M., Golenbock D.T., Kummer M.P. (2015). Neuroinflammation in Alzheimer's disease. Lancet Neurol..

[10] Heppner F.L., Ransohoff R.M., Becher B. (2015). Immune attack: the role of inflammation in Alzheimer disease. Nat. Rev. Neurosci..

[11] Morgan B.P. (2018). Complement in the pathogenesis of Alzheimer's disease. Semin. Immunopathol..

[12] Giasson B.I., Ischiropoulos H., Lee V.M.Y., Trojanowski J.Q. (2002). The relationship between oxidative/nitrative stress and pathological inclusions in Alzheimer's and Parkinson's diseases. Free Radic. Biol. Med..

[13] Souza J.M., Peluffo G., Radi R. (2008). Protein tyrosine nitration--functional alteration or just a biomarker?. Free Radic. Biol. Med..

[14] Carvalho C., Moreira P.I. (2018). Oxidative stress: a major player in cerebrovascular alterations associated to neurodegenerative events. Front. Physiol..

[15] Butterfield D.A., Halliwell B. (2019). Oxidative stress, dysfunctional glucose metabolism and Alzheimer disease. Nat. Rev. Neurosci..

[16] Cai Q., Tammineni P. (2017). Mitochondrial aspects of synaptic dysfunction in Alzheimer's disease. J. Alzheimers Dis..

[17] Blokzijl F., de Ligt J., Jager M., Sasselli V., Roerink S., Sasaki N., Huch M., Boymans S., Kuijk E., Prins P., Nijman I.J., Martincorena I., Mokry M., Wiegerinck C.L., Middendorp S., Sato T., Schwank G., Nieuwenhuis E.E., Verstegen M.M., van der Laan L.J., de Jonge J., Jn I.J., Vries R.G., van de Wetering M., Stratton M.R., Clevers H., Cuppen E., van Boxtel R. (2016). Tissue-specific mutation accumulation in human adult stem cells during life. Nature.

[18] Miller M.B., Huang A.Y., Kim J., Zhou Z., Kirkham S.L., Maury E.A., Ziegenfuss J.S., Reed H.C., Neil J.E., Rento L., Ryu S.C., Ma C.C., Luquette L.J., Ames H.M., Oakley D.H., Frosch M.P., Hyman B.T., Lodato M.A., Lee E.A., Walsh C.A. (2022). Somatic genomic changes in single Alzheimer's disease neurons. Nature.

[19] Bush A.I. (2003). The metallobiology of Alzheimer's disease. Trends Neurosci..

[20] Milton N.G. (2004). Role of hydrogen peroxide in the aetiology of Alzheimer's disease: implications for treatment. Drugs Aging.

[21] Tabner B.J., El-Agnaf O.M., Turnbull S., German M.J., Paleologou K.E., Hayashi Y., Cooper L.J., Fullwood N.J., Allsop D. (2005). Hydrogen peroxide is generated during the very early stages of aggregation of the amyloid peptides implicated in Alzheimer disease and familial British dementia. J. Biol. Chem..

[22] Akama K.T., Albanese C., Pestell R.G., Van Eldik L.J. (1998). Amyloid beta-peptide stimulates nitric oxide production in astrocytes through an NFkappaB-dependent mechanism. Proc. Natl. Acad. Sci. U. S. A..

[23] Loboda A., Damulewicz M., Pyza E., Jozkowicz A., Dulak J. (2016). Role of Nrf2/HO-1 system in development, oxidative stress response and diseases: an evolutionarily conserved mechanism. Cell. Mol. Life Sci..

[24] Crawford F.C., Freeman M.J., Schinka J.A., Morris M.D., Abdullah L.I., Richards D., Sevush S., Duara R., Mullan M.J. (2001). Association between Alzheimer's disease and a functional polymorphism in the Myeloperoxidase gene. Exp. Neurol..

[25] Green P.S., Mendez A.J., Jacob J.S., Crowley J.R., Growdon W., Hyman B.T., Heinecke J.W. (2004). Neuronal expression of myeloperoxidase is increased in Alzheimer's disease. J. Neurochem..

[26] Palaniyappan A., Uwiera R.R., Idikio H., Jugdutt B.I. (2009). Comparison of vasopeptidase inhibitor omapatrilat and angiotensin receptor blocker candesartan on extracellular matrix, myeloperoxidase, cytokines, and ventricular remodeling during healing after reperfused myocardial infarction. Mol. Cell. Biochem..

[27] Radi R. (2004). Nitric oxide, oxidants, and protein tyrosine nitration. Proc. Natl. Acad. Sci. U. S. A..

[28] Reynolds M.R., Reyes J.F., Fu Y., Bigio E.H., Guillozet-Bongaarts A.L., Berry R.W., Binder L.I. (2006). Tau nitration occurs at tyrosine 29 in the fibrillar lesions of Alzheimer's disease and other tauopathies. J. Neurosci..

[29] Good P.F., Werner P., Hsu A., Olanow C.W., Perl D.P. (1996). Evidence of neuronal oxidative damage in Alzheimer's disease. Am. J. Pathol..

[30] Hensley K., Maidt M.L., Yu Z., Sang H., Markesbery W.R., Floyd R.A. (1998). Electrochemical analysis of protein nitrotyrosine and dityrosine in the Alzheimer brain indicates region-specific accumulation. J. Neurosci..

[31] Luth H.J., Munch G., Arendt T. (2002). Aberrant expression of NOS isoforms in Alzheimer's disease is structurally related to nitrotyrosine formation. Brain Res..

[32] Castegna A., Thongboonkerd V., Klein J.B., Lynn B., Markesbery W.R., Butterfield D.A. (2003). Proteomic identification of nitrated proteins in Alzheimer's disease brain. J. Neurochem..

[33] Calabrese V., Sultana R., Scapagnini G., Guagliano E., Sapienza M., Bella R., Kanski J., Pennisi G., Mancuso C., Stella A.M., Butterfield D.A. (2006). Nitrosative stress, cellular stress response, and thiol homeostasis in patients with Alzheimer's disease. Antioxidants Redox Signal..

[34] Butterfield D.A., Reed T.T., Perluigi M., De Marco C., Coccia R., Keller J.N., Markesbery W.R., Sultana R. (2007). Elevated levels of 3-nitrotyrosine in brain from subjects with amnestic mild cognitive impairment: implications for the role of nitration in the progression of Alzheimer's disease. Brain Res..

[35] Sultana R., Reed T., Perluigi M., Coccia R., Pierce W.M., Butterfield D.A. (2007). Proteomic identification of nitrated brain proteins in amnestic mild cognitive impairment: a regional study. J. Cell Mol. Med..

[36] Tohgi H., Abe T., Yamazaki K., Murata T., Ishizaki E., Isobe C. (1999). Alterations of 3-nitrotyrosine concentration in the cerebrospinal fluid during aging and in patients with Alzheimer's disease. Neurosci. Lett..

[37] Sultana R., Poon H.F., Cai J., Pierce W.M., Merchant M., Klein J.B., Markesbery W.R., Butterfield D.A. (2006). Identification of nitrated proteins in Alzheimer's disease brain using a redox proteomics approach. Neurobiol. Dis..

[38] Reed T.T., Pierce W.M., Turner D.M., Markesbery W.R., Butterfield D.A. (2009). Proteomic identification of nitrated brain proteins in early Alzheimer's disease inferior parietal lobule. J. Cell Mol. Med..

[39] Butterfield D.A., Perluigi M., Sultana R. (2006). Oxidative stress in Alzheimer's disease brain: new insights from redox proteomics. Eur. J. Pharmacol..

[40] Sultana R., Poon H.F., Cai J., Pierce W.M., Merchant M., Klein J.B., Markesbery W.R., Butterfield D.A. (2006). Identification of nitrated proteins in Alzheimer's disease brain using a redox proteomics approach. Neurobiol. Dis..

[41] Milton N.N. (2004). Role of hydrogen peroxide in the aetiology of Alzheimer's disease. Drugs Aging.

[42] Butterfield D.A., Reed T., Sultana R. (2011). Roles of 3-nitrotyrosine- and 4-hydroxynonenal-modified brain proteins in the progression and pathogenesis of Alzheimer's disease. Free Radic. Res..

[43] Butterfield D.A., Reed T., Newman S.F., Sultana R. (2007). Roles of amyloid beta-peptide-associated oxidative stress and brain protein modifications in the pathogenesis of Alzheimer's disease and mild cognitive impairment. Free Radic. Biol. Med..

[44] Braak H., Braak E. (1997). Frequency of stages of Alzheimer-related lesions in different age categories. Neurobiol. Aging.

[45] Ashby E.L., Kierzkowska M., Hull J., Kehoe P.G., Hutson S.M., Conway M.E. (2017). Altered expression of human mitochondrial branched chain aminotransferase in dementia with lewy bodies and vascular dementia. Neurochem. Res..

[46] Kehoe P.G., Wong S., Al Mulhim N., Palmer L.E., Miners J.S. (2016). Angiotensin-converting enzyme 2 is reduced in Alzheimer's disease in association with increasing amyloid-β and tau pathology. Alzheimers Res. Ther..

[47] Montine T.J., Phelps C.H., Beach T.G., Bigio E.H., Cairns N.J., Dickson D.W., Duyckaerts C., Frosch M.P., Masliah E., Mirra S.S., Nelson P.T., Schneider J.A., Thal D.R., Trojanowski J.Q., Vinters H.V., Hyman B.T. (2012). National Institute on Aging-Alzheimer's Association guidelines for the neuropathologic assessment of Alzheimer's disease: a practical approach. Acta Neuropathol..

[48] DeTure M.A., Dickson D.W. (2019). The neuropathological diagnosis of Alzheimer's disease. Mol. Neurodegener..

[49] Mirra S.S., Heyman A., McKeel D., Sumi S.M., Crain B.J., Brownlee L.M., Vogel F.S., Hughes J.P., van Belle G., Berg L. (1991). The consortium to establish a Registry for Alzheimer's disease (CERAD). Part II. Standardization of the neuropathologic assessment of Alzheimer's disease. Neurology.

[50] Braak H., Braak E. (1991). Neuropathological stageing of Alzheimer-related changes. Acta Neuropathol..

[51] Chalmers K., Wilcock G.K., Love S. (2003). APOE epsilon 4 influences the pathological phenotype of Alzheimer's disease by favouring cerebrovascular over parenchymal accumulation of A beta protein. Neuropathol. Appl. Neurobiol..

[52] Drabkin D.L. (1949). The standardization of hemoglobin measurement. Am. J. Med. Sci..

[53] Shevchenko A., Tomas H., Havlis J., Olsen J.V., Mann M. (2006). In-gel digestion for mass spectrometric characterization of proteins and proteomes. Nat. Protoc..

[54] MatrixScience (2014). Matrix science - Mascot - peptide mass fingerprint. http://www.matrixscience.com/help/interpretation_help.html.

[55] Consortium T.U. (2015). UniProt: a hub for protein information. Nucleic Acids Res..

[56] Knight A.R., Taylor E.L., Lukaszewski R., Jensen K.T., Jones H.E., Carre J.E., Isupov M.N., Littlechild J.A., Bailey S.J., Brewer E., McDonald T.J., Pitt A.R., Spickett C.M., Winyard P.G. (2018). A high-sensitivity electrochemiluminescence-based ELISA for the measurement of the oxidative stress biomarker, 3-nitrotyrosine, in human blood serum and cells. Free Radic. Biol. Med..

[57] Higuchi K., Motomizu S. (1999). Flow-injection spectrophotometric determination of nitrite and nitrate in biological samples. Anal. Sci..

[58] Bateman R.M., Ellis C.G., Freeman D.J. (2002). Optimization of nitric oxide chemiluminescence operating conditions for measurement of plasma nitrite and nitrate. Clin. Chem..

[59] Squier T.C. (2001). Oxidative stress and protein aggregation during biological aging. Exp. Gerontol..

[60] Denicola A., Souza J.M., Radi R. (1998). Diffusion of peroxynitrite across erythrocyte membranes. Proc. Natl. Acad. Sci. U. S. A..

[61] Htun N.M., Chen Y.C., Lim B., Schiller T., Maghzal G.J., Huang A.L., Elgass K.D., Rivera J., Schneider H.G., Wood B.R., Stocker R., Peter K. (2017). Near-infrared autofluorescence induced by intraplaque hemorrhage and heme degradation as marker for high-risk atherosclerotic plaques. Nat. Commun..

[62] Butterfield D.A., Poon H.F., Clair D. St, Keller J.N., Pierce W.M., Klein J.B., Markesbery W.R. (2006). Redox proteomics identification of oxidatively modified hippocampal proteins in mild cognitive impairment: insights into the development of Alzheimer's disease. Neurobiol. Dis..

[63] Romero N., Radi R., Linares E., Augusto O., Detweiler C.D., Mason R.P., Denicola A. (2003). Reaction of human hemoglobin with peroxynitrite. Isomerization to nitrate and secondary formation of protein radicals. J. Biol. Chem..

[64] Richter F., Meurers B.H., Zhu C., Medvedeva V.P., Chesselet M.F. (2009). Neurons express hemoglobin alpha- and beta-chains in rat and human brains. J. Comp. Neurol..

[65] Hundahl C.A., Kelsen J., Hay-Schmidt A. (2013). Neuroglobin and cytoglobin expression in the human brain. Brain Struct. Funct..

[66] De Reuck J.L. (2012). The significance of small cerebral bleeds in neurodegenerative dementia syndromes. Aging Dis..

[67] Govindpani K., McNamara L.G., Smith N.R., Vinnakota C., Waldvogel H.J., Faull R.L., Kwakowsky A. (2019). Vascular dysfunction in Alzheimer's disease: a prelude to the pathological process or a consequence of it?. J. Clin. Med..

[68] Slemmon J.R., Hughes C.M., Campbell G.A., Flood D.G. (1994). Increased levels of hemoglobin-derived and other peptides in Alzheimer's disease cerebellum. J. Neurosci..

[69] O'Callaghan J., Holmes H., Powell N., Wells J.A., Ismail O., Harrison I.F., Siow B., Johnson R., Ahmed Z., Fisher A., Meftah S., O'Neill M.J., Murray T.K., Collins E.C., Shmueli K., Lythgoe M.F. (2017). Tissue magnetic susceptibility mapping as a marker of tau pathology in Alzheimer's disease. Neuroimage.

[70] Oh J.Y., Williams A., Patel R.P. (2019). The role of redox-dependent mechanisms in heme release from hemoglobin and erythrocyte hemolysates. Arch. Biochem. Biophys..

[71] Si Z., Wang X. (2020). The neuroprotective and neurodegeneration effects of heme oxygenase-1 in Alzheimer's disease. J. Alzheimers Dis..

[72] Halliwell B. (2001). Role of free radicals in the neurodegenerative diseases. Drugs Aging.

[73] Ye Z., Ander B.P., Sharp F.R., Zhan X. (2018). Cleaved β-actin may contribute to DNA fragmentation following very brief focal cerebral ischemia. J. Neuropathol. Exp. Neurol..

[74] Takahashi N., Hayano T., Suzuki M. (1989). Peptidyl-prolyl cis-trans isomerase is the cyclosporin A-binding protein cyclophilin. Nature.

[75] Wang L., Zhou Y., Chen D., Lee T.H. (2020). Peptidyl-prolyl cis/trans isomerase Pin1 and Alzheimer's disease. Front. Cell Dev. Biol..

[76] Bell R.D., Winkler E.A., Singh I., Sagare A.P., Deane R., Wu Z., Holtzman D.M., Betsholtz C., Armulik A., Sallstrom J., Berk B.C., Zlokovic B.V. (2012). Apolipoprotein E controls cerebrovascular integrity via cyclophilin A. Nature.

[77] Castegna A., Aksenov M., Thongboonkerd V., Klein J.B., Pierce W.M., Booze R., Markesbery W.R., Butterfield D.A. (2002). Proteomic identification of oxidatively modified proteins in Alzheimer's disease brain. Part II: dihydropyrimidinase-related protein 2, alpha-enolase and heat shock cognate 71. J. Neurochem..

[78] Bunn H.F., Gabbay K.H., Gallop P.M. (1978). The glycosylation of hemoglobin: relevance to diabetes mellitus. Science.

[79] McKhann G.M., Knopman D.S., Chertkow H., Hyman B.T., Jack C.R., Kawas C.H., Klunk W.E., Koroshetz W.J., Manly J.J., Mayeux R., Mohs R.C., Morris J.C., Rossor M.N., Scheltens P., Carrillo M.C., Thies B., Weintraub S., Phelps C.H. (2011). The diagnosis of dementia due to Alzheimer's disease: recommendations from the National Institute on Aging-Alzheimer’s Association workgroups on diagnostic guidelines for Alzheimer's disease. Alzheimer's Dementia.

[80] Blennow K., Hampel H., Weiner M., Zetterberg H. (2010). Cerebrospinal fluid and plasma biomarkers in Alzheimer disease. Nat. Rev. Neurol..

[81] Ryu J.K., McLarnon J.G. (2009). A leaky blood-brain barrier, fibrinogen infiltration and microglial reactivity in inflamed Alzheimer's disease brain. J. Cell Mol. Med..

[82] Zipser B.D., Johanson C.E., Gonzalez L., Berzin T.M., Tavares R., Hulette C.M., Vitek M.P., Hovanesian V., Stopa E.G. (2007). Microvascular injury and blood-brain barrier leakage in Alzheimer's disease. Neurobiol. Aging.

[83] Banks W.A., Kovac A., Majerova P., Bullock K.M., Shi M., Zhang J. (2017). Tau proteins cross the blood-brain barrier. J. Alzheimers Dis..

[84] Nirmalraj P.N., Schneider T., Felbecker A. (2021). Spatial organization of protein aggregates on red blood cells as physical biomarkers of Alzheimer's disease pathology. Sci. Adv..

[85] Lv H., Wei G.Y., Guo C.S., Deng Y.F., Jiang Y.M., Gao C., Jian C.D. (2020). 20S proteasome and glyoxalase 1 activities decrease in erythrocytes derived from Alzheimer's disease patients. Neural Regen. Res..

[86] Palmieri G., Cocca E., Gogliettino M., Valentino R., Ruvo M., Cristofano G., Angiolillo A., Balestrieri M., Rossi M., Di Costanzo A. (2017). Low erythrocyte levels of proteasome and acyl-peptide hydrolase (APEH) activities in Alzheimer's disease: a sign of defective proteostasis?. J. Alzheimers Dis..

[87] Sharma A., Weber D., Raupbach J., Dakal T.C., Fließbach K., Ramirez A., Grune T., Wüllner U. (2020). Advanced glycation end products and protein carbonyl levels in plasma reveal sex-specific differences in Parkinson's and Alzheimer's disease. Redox Biol..

[88] Vignini A., Giusti L., Raffaelli F., Giulietti A., Salvolini E., Luzzi S., Provinciali L., Mazzanti L., Nanetti L. (2013). Impact of gender on platelet membrane functions of Alzheimer's disease patients. Exp. Gerontol..

[89] Radi R. (2013). Protein tyrosine nitration: biochemical mechanisms and structural basis of functional effects. Acc. Chem. Res..

[90] Schaer D.J., Buehler P.W., Alayash A.I., Belcher J.D., Vercellotti G.M. (2013). Hemolysis and free hemoglobin revisited: exploring hemoglobin and hemin scavengers as a novel class of therapeutic proteins. Blood.

[91] Potor L., Hendrik Z., Patsalos A., Katona É., Méhes G., Póliska S., Csősz É., Kalló G., Komáromi I., Combi Z., Posta N., Sikura K.É., Pethő D., Oros M., Vereb G., Tóth C., Gergely P., Nagy L., Balla G., Balla J. (2021). Oxidation of hemoglobin drives a proatherogenic polarization of macrophages in human atherosclerosis. Antioxidants Redox Signal..

[92] Masuoka N., Sugiyama H., Ishibashi N., Wang D.H., Masuoka T., Kodama H., Nakano T. (2006). Characterization of acatalasemic erythrocytes treated with low and high dose hydrogen peroxide. Hemolysis and aggregation. J. Biol. Chem..

[93] Reeder B.J., Sharpe M.A., Kay A.D., Kerr M., Moore K., Wilson M.T. (2002). Toxicity of myoglobin and haemoglobin: oxidative stress in patients with rhabdomyolysis and subarachnoid haemorrhage. Biochem. Soc. Trans..

[94] Chen H.J., Chen Y.C. (2012). Reactive nitrogen oxide species-induced post-translational modifications in human hemoglobin and the association with cigarette smoking. Anal. Chem..

[95] Giulivi C., Davies K.J. (2001). Mechanism of the formation and proteolytic release of H2O2-induced dityrosine and tyrosine oxidation products in hemoglobin and red blood cells. J. Biol. Chem..

[96] Grzelak A., Balcerczyk A., Mateja A., Bartosz G. (2001). Hemoglobin can nitrate itself and other proteins. Biochim. Biophys. Acta.

[97] Chen H.J., Yang Y.F., Lai P.Y., Chen P.F. (2016). Analysis of chlorination, nitration, and nitrosylation of tyrosine and oxidation of methionine and cysteine in hemoglobin from type 2 diabetes mellitus patients by nanoflow liquid chromatography tandem mass spectrometry. Anal. Chem..

[98] Altinoz M.A., Guloksuz S., Schmidt-Kastner R., Kenis G., Ince B., Rutten B.P.F. (2019). Involvement of hemoglobins in the pathophysiology of Alzheimer's disease. Exp. Gerontol..

[99] Drvenica I.T., Stančić A.Z., Maslovarić I.S., Trivanović D.I., Ilić V.L. (2022). Extracellular hemoglobin: modulation of cellular functions and pathophysiological effects. Biomolecules.

[100] Chen G.-f., Xu T.-h., Yan Y., Zhou Y.-r., Jiang Y., Melcher K., Xu H.E. (2017). Amyloid beta: structure, biology and structure-based therapeutic development. Acta Pharmacol. Sin..

[101] Chuang J.Y., Lee C.W., Shih Y.H., Yang T., Yu L., Kuo Y.M. (2012). Interactions between amyloid-beta and hemoglobin: implications for amyloid plaque formation in Alzheimer's disease. PLoS One.

[102] Wu C.-W., Liao P.-C., Yu L., Wang S.-T., Chen S.-T., Wu C.-M., Kuo Y.-M. (2004). Hemoglobin promotes Aβ oligomer formation and localizes in neurons and amyloid deposits. Neurobiol. Dis..

[103] Kummer M.P., Hermes M., Delekarte A., Hammerschmidt T., Kumar S., Terwel D., Walter J., Pape H.C., Konig S., Roeber S., Jessen F., Klockgether T., Korte M., Heneka M.T. (2011). Nitration of tyrosine 10 critically enhances amyloid beta aggregation and plaque formation. Neuron.

[104] Ghosh C., Seal M., Mukherjee S., Ghosh Dey S. (2015). Alzheimer's disease: a heme-abeta perspective. Acc. Chem. Res..

[105] Yuan C., Gao Z. (2013). Abeta interacts with both the iron center and the porphyrin ring of heme: mechanism of heme's action on Abeta aggregation and disaggregation. Chem. Res. Toxicol..

[106] Thiabaud G., Pizzocaro S., Garcia-Serres R., Latour J.M., Monzani E., Casella L. (2013). Heme binding induces dimerization and nitration of truncated beta-amyloid peptide Abeta16 under oxidative stress. Angew Chem. Int. Ed. Engl..

[107] Ghosh C., Mukherjee S., Seal M., Dey S.G. (2016). Peroxidase to cytochrome b type transition in the active site of heme-bound amyloid β peptides relevant to Alzheimer's disease. Inorg. Chem..

[108] Nath A.K., Ghosh C., Roy M., Seal M., Ghosh S. (2019). Nitrite reductase activity of heme and copper bound Aβ peptides. Dalton Trans..

[109] Nath A.K., Roy M., Dey C., Dey A., Dey S.G. (2022). Spin state dependent peroxidase activity of heme bound amyloid β peptides relevant to Alzheimer's disease. Chem. Sci..

[110] Pal I., Dey S.G. (2023). The role of heme and copper in Alzheimer's disease and type 2 diabetes mellitus. JACS Au.

[111] Jayakumar R., Kusiak J.W., Chrest F.J., Demehin A.A., Murali J., Wersto R.P., Nagababu E., Ravi L., Rifkind J.M. (2003). Red cell perturbations by amyloid beta-protein. Biochim. Biophys. Acta.

[112] Cheng J., North B.J., Zhang T., Dai X., Tao K., Guo J., Wei W. (2018). The emerging roles of protein homeostasis-governing pathways in Alzheimer's disease. Aging Cell.

[113] Thibaudeau T.A., Smith D.M. (2019). A practical review of proteasome pharmacology. Pharmacol. Rev..

[114] Harris L.D., Jasem S., Licchesi J.D.F. (2020). The ubiquitin system in alzheimer's disease. Adv. Exp. Med. Biol..

[115] Souza J.M., Choi I., Chen Q., Weisse M., Daikhin E., Yudkoff M., Obin M., Ara J., Horwitz J., Ischiropoulos H. (2000). Proteolytic degradation of tyrosine nitrated proteins. Arch. Biochem. Biophys..

[116] Keller J.N., Hanni K.B., Markesbery W.R. (2000). Impaired proteasome function in Alzheimer's disease. J. Neurochem..

[117] López Salon M., Morelli L., Castaño E.M., Soto E.F., Pasquini J.M. (2000). Defective ubiquitination of cerebral proteins in Alzheimer's disease. J. Neurosci. Res..

[118] Tramutola A., Lanzillotta C., Perluigi M., Butterfield D.A. (2017). Oxidative stress, protein modification and Alzheimer disease. Brain Res. Bull..

[119] Zheng Q., Huang T., Zhang L., Zhou Y., Luo H., Xu H., Wang X. (2016). Dysregulation of ubiquitin-proteasome system in neurodegenerative diseases. Front. Aging Neurosci..

[120] Smallwood M.J., Nissim A., Knight A.R., Whiteman M., Haigh R., Winyard P.G. (2018). Oxidative stress in autoimmune rheumatic diseases. Free Radic. Biol. Med..

[121] Lim B., Prassas I., Diamandis E.P. (2020). Alzheimer disease pathogenesis: the role of autoimmunity. J. Appl. Lab. Med..

[122] Wong V.W., Lerner E. (2015). Nitric oxide inhibition strategies. Future Sci. OA.

[123] Lee S.J., Kim T.U., Park J.S., Ra J.Y. (2013). Inhibition of nitric oxide mediated protein nitration: therapeutic implications in experimental radiculopathy. Spine.

[124] Zielonka J., Zielonka M., VerPlank L., Cheng G., Hardy M., Ouari O., Ayhan M.M., Podsiadly R., Sikora A., Lambeth J.D., Kalyanaraman B. (2016). Mitigation of NADPH oxidase 2 activity as a strategy to inhibit peroxynitrite formation. J. Biol. Chem..

[125] Diebold B.A., Smith S.M., Li Y., Lambeth J.D. (2015). NOX2 as a target for drug development: indications, possible complications, and progress. Antioxidants Redox Signal..

[126] Aoyama T., Paik Y.H., Watanabe S., Laleu B., Gaggini F., Fioraso-Cartier L., Molango S., Heitz F., Merlot C., Szyndralewiez C., Page P., Brenner D.A. (2012). Nicotinamide adenine dinucleotide phosphate oxidase in experimental liver fibrosis: GKT137831 as a novel potential therapeutic agent. Hepatology.

[127] Tiden A.K., Sjogren T., Svensson M., Bernlind A., Senthilmohan R., Auchere F., Norman H., Markgren P.O., Gustavsson S., Schmidt S., Lundquist S., Forbes L.V., Magon N.J., Paton L.N., Jameson G.N., Eriksson H., Kettle A.J. (2011). 2-thioxanthines are mechanism-based inactivators of myeloperoxidase that block oxidative stress during inflammation. J. Biol. Chem..

