# Supplementary material for: Hemoglobin in the brain frontal lobe tissue of patients with Alzheimer’s disease is susceptible to reactive nitrogen species-mediated oxidative damage
Source: Redox Biol. 2025 Mar 25;82:103612. doi: 10.1016/j.redox.2025.103612 (PMC11999687; doi:10.1016/j.redox.2025.103612)
Supplement: Multimedia component 1 [file mmc1.docx]

**Supplementary Material**

**Hemoglobin in the brain frontal lobe tissue of patients with Alzheimer’s disease is susceptible to reactive nitrogen species-mediated oxidative damage**

M.J. Smallwood^a^, M. Abu Alghayth^a,f^, A.R. Knight^a^, K. Tveen-Jensen^b^, A.R. Pitt^b^, C.M. Spickett^b^, D. Llewellyn^a^, G. Pula^a,c^, A.R. Wearn^d^, A. Vanhatalo^a^, A.M. Jones^a^, P. Francis^a,e^, E. Coulthard^d^, P. G. Kehoe^d^ and P.G. Winyard^a^

*^a^ University of Exeter Medical School, Exeter, EX1 2LU, UK*

*^b^ College of Health & Life Sciences, Aston University, Birmingham, B4 7ET, UK*

^c^ *Centre for Biomedicine, Hull York Medical School, Hull, HU6 7RX, UK*

*^d^ Bristol Medical School, University of Bristol, Southmead Hospital, Bristol, BS10 5NB, UK*

*^e^ Institute of Psychiatry, Psychology and Neuroscience, King’s College, University of London, London, WC2R 2LS, UK*

*^f^ Current address: Department of Medical Laboratory Sciences, College of Applied Medical Sciences, University of Bisha, Bisha 67714, P.O. Box 255, Saudi Arabia*

**Supplementary Table 1, Part A.**

| **Proteins identified** | **Protein mass (Da)** | **Ion Score** | **emPAI score** | **Confidence score** |
| --- | --- | --- | --- | --- |
| **Spectrin-β, non-erythrocytic** | **274,439** | **8,240** | **3.59** | **3** |
| Pyruvate kinase | 57,900 | 3,742 | 2.76 | 2 |
| **Spectrin-α, non-erythrocytic** | **284,364** | **3,654** | **1.46** | **3** |
| Serum albumin | 69,321 | 20,012 | 1.41 | 2 |
| Keratin type II cytoskeletal | 65,999 | 689 | 0.97 | 0 |
| Hemoglobin-α | 15,248 | 107 | 0.83 | 1 |
| Creatine kinase | 42,617 | 1,751 | 0.82 | 1 |
| Myelin proteolipid protein | 30,057 | 259 | 0.69 | 1 |
| Dihydropyrimidinase-related protein 2 | 62,255 | 626 | 0.67 | 1 |
| V-type proton ATPase | 56,465 | 544 | 0.57 | 1 |

**Supplementary Table 1, Part B.**

| **Proteins identified** | **Protein mass (Da)** | **Ion Score** | **emPAI score** | **Confidence score** |
| --- | --- | --- | --- | --- |
| **Spectrin-β, non-erythrocytic** | **274,439** | **7,710** | **3.32** | **3** |
| Hemoglobin-β | 15,988 | 165 | 2.17 | 1 |
| Myelin proteolipid protein | 30,057 | 652 | 1.57 | 1 |
| Tubulin-β4A | 49,554 | 809 | 1.47 | 2 |
| Tubulin-β | 49,639 | 776 | 1.46 | 2 |
| Tubulin-β2A | 49,875 | 738 | 1.3 | 1 |
| Tubulin-α4A | 49,892 | 497 | 1.3 | 1 |
| Tubulin-α1A | 50,104 | 551 | 1.29 | 0 |
| Hemoglobin-α | 15,248 | 127 | 1.24 | 1 |
| Sodium/potassium-transporting ATPase | 112,824 | 1,334 | 1.1 | 2 |

**Supplementary Table 1, Part C.**

| **Proteins identified** | **Protein mass (Da)** | **Ion Score** | **emPAI score** | **Confidence score** |
| --- | --- | --- | --- | --- |
| **Spectrin-β, non-erythrocytic** | **274,439** | **6,271** | **4.16** | **3** |
| Hemoglobin-β | 15,988 | 147 | 2.84 | 1 |
| **Spectrin-α, non-erythrocytic** | **284,364** | **3,186** | **1.82** | **3** |
| Hemoglobin-α | 15,248 | 156 | 1.24 | 1 |
| Serum albumin | 69,321 | 2,928 | 1 | 2 |
| Keratin type II cytoskeletal | 65,999 | 433 | 0.79 | 0 |
| Keratin type I cytoskeletal | 62,027 | 339 | 0.76 | 0 |
| Myelin proteolipid protein | 30,057 | 227 | 0.69 | 1 |
| Tubulin-α1B | 50,120 | 83 | 0.37 | 1 |
| Keratin type II cytoskeletal 2 | 65,393 | 154 | 0.34 | 0 |

**Supplementary Table 1, Part D.**

| **Proteins identified** | **Protein mass (Da)** | **Ion Score** | **emPAI** | **Confidence score** |
| --- | --- | --- | --- | --- |
| **Pyruvate kinase** | **57,900** | **16,908** | **29.71** | **3** |
| Rab GDP dissociation inhibitor | 50550 | 6,336 | 22.6 | 2 |
| **Dihydropyrimidinase-related protein 2** | **62,255** | **7,730** | **12.1** | **3** |
| V-type proton ATPase | 56,465 | 3,254 | 6.26 | 2 |
| Hemoglobin-β | 15,988 | 235 | 5.85 | 1 |
| Hemoglobin-α | 15,248 | 382 | 3.1 | 1 |
| Syntaxin-binding protein 1 | 67,526 | 1,974 | 2.61 | 2 |
| **Glucose-6-phosphate isomerase** | **63,107** | **2,461** | **2.56** | **3** |
| **Heat shock protein (60 kDa)** | **61,016** | **1,795** | **2.52** | **3** |
| Alpha-aminoadipic semialdehyde dehydrogenase | 58,450 | 984 | 2.52 | 2 |

**Supplementary Table 1, Part E.**

| **Proteins identified** | **Protein mass (Da)** | **Ion Score** | **emPAI score** | **Confidence score** |
| --- | --- | --- | --- | --- |
| **Peptidyl-prolyl *cis-trans* isomerase** | **18,001** | **4,457** | **43.03** | **3** |
| Histone H2B type 1C | 13,898 | 1,429 | 8.01 | 2 |
| Histone H2B type 1B | 13,942 | 1,358 | 7.86 | 2 |
| Hemoglobin-β | 15,988 | 341 | 7.3 | 1 |
| Peroxiredoxin-V | 22,073 | 1,443 | 4.45 | 2 |
| Nucleoside diphosphate kinase | 17,287 | 494 | 3.16 | 2 |
| Visinin-like protein | 22,128 | 355 | 3.11 | 1 |
| Gamma-synuclein | 13,323 | 291 | 2.94 | 1 |
| Destrin | 18,493 | 243 | 2.81 | 2 |
| Tubulin-β2A | 49,875 | 1,108 | 2.38 | 2 |

**Supplementary Table 1, Part F.**

| **Proteins identified** | **Protein mass (Da)** | **Ion Score** | **emPAI score** | **Confidence score** |
| --- | --- | --- | --- | --- |
| **Hemoglobin-β** | **15,988** | **14,423** | **179.23** | **3** |
| Hemoglobin-δ | 16,045 | 9,272 | 36.76 | 2 |
| **Hemoglobin-α** | **15,248** | **7,804** | **36.7** | **3** |
| Histone H4 | 11,360 | 1,018 | 9.93 | 2 |
| Fatty acid binding protein | 14,849 | 760 | 5.38 | 2 |
| Cytochrome C | 11,741 | 507 | 5.02 | 1 |
| Profilin-1 | 15,045 | 406 | 3.14 | 1 |
| Cystatin B | 11,133 | 378 | 2.87 | 1 |
| Galectin 1 | 14,706 | 364 | 2.5 | 2 |
| Profilin-2 | 15,036 | 654 | 2.41 | 2 |

**Supplementary Table 1, Part G.**

| **Proteins identified** | **Protein mass (Da)** | **Ion Score** | **emPAI score** | **Confidence score** |
| --- | --- | --- | --- | --- |
| **Hemoglobin-β** | **15,988** | **11,795** | **217.46** | **3** |
| Hemoglobin-δ | 16,045 | 7,201 | 44.72 | 2 |
| **Hemoglobin-α** | **15,248** | **6,266** | **24.19** | **3** |
| Histone H4 | 11,360 | 1,132 | 23.24 | 2 |
| Cytochrome b-c1 complex | 13,522 | 288 | 5.04 | 1 |
| Cytochrome c oxidase | 8,776 | 76 | 4.36 | 1 |
| Cytochrome c | 11,741 | 362 | 3.66 | 1 |
| Hemoglobin-γ1 | 16,130 | 1,082 | 3.61 | 2 |
| Hemoglobin-γ2 | 16,116 | 1,036 | 3.61 | 2 |
| Galectin 1 | 14,706 | 378 | 2.5 | 2 |

Supplementary Table 1. The ten most abundant proteins by exponentially modified protein abundance index (emPAI) score identified by mass spectrometry in bands excised from the Coomassie-stained gel of Alzheimer’s disease and non-dementia brain samples. The Coomassie-stained gel from which bands were excised and identified, is shown in Supplementary Fig. 1. Parts A, B and C of the table show that the predominant protein identified by mass spectrometry in bands A, B and C (the bands at approximately 300 kDa in Supplementary Fig. 1) was non-erythrocytic spectrin-β. Part D of the table shows that band D (the 60 kDa band in Supplementary Fig. 1) contained predominantly pyruvate kinase and dihydropyrimidinase-related protein 2. Part E of the table shows that band E (Supplementary Fig. 2) contained peptidyl-propyl *cis-trans* isomerase. Parts F and G of the table show hemoglobin-α and hemoglobin-β as the predominant proteins identified by mass spectrometry. These proteins are organized according to their emPAI score [1]. Proteins with a confidence score of 3 are shown in bold text and proteins with a confidence score of 0 are shown in grey font. Text that is neither bold nor grey corresponds to scores 1 and 2.


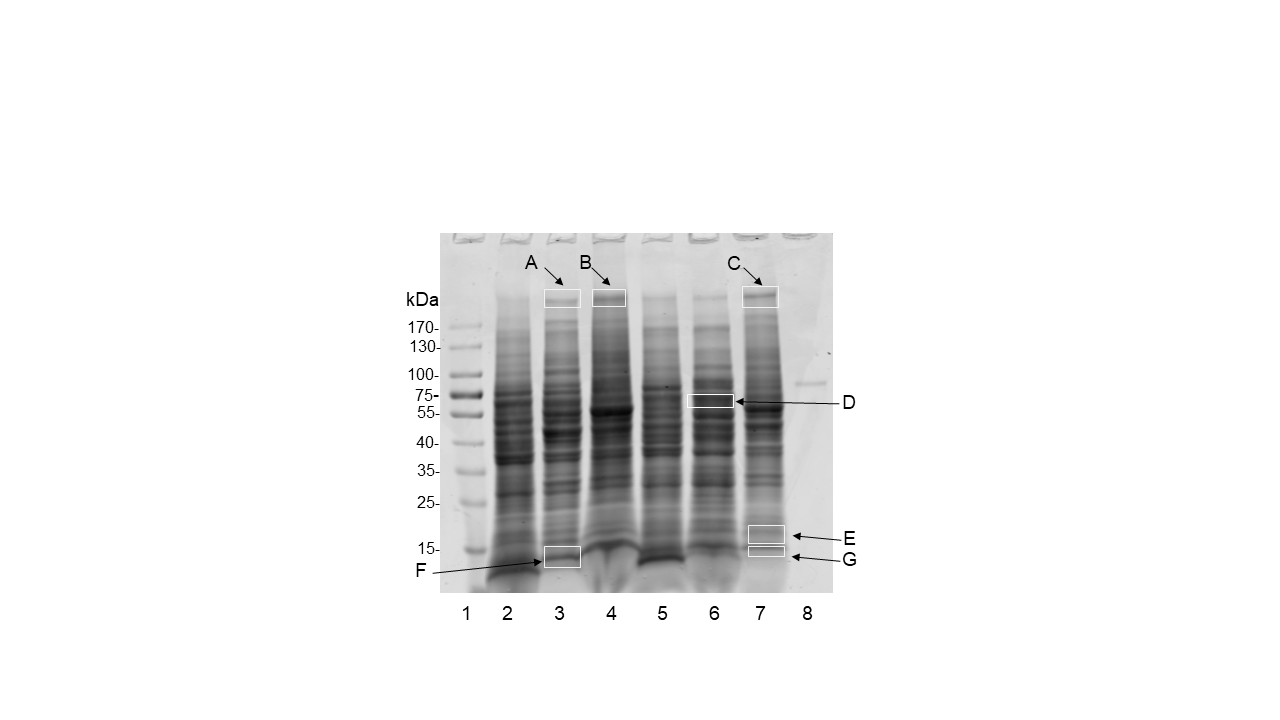


**Supplementary Fig. 1 Coomassie-stained gel in which brain tissue extracts from Alzheimer’s disease, vascular dementia patients and non-dementia controls were electrophoresed, showing the location of protein bands before they were excised.** Protein bands were separated by 12% SDS-polyacrylamide gel electrophoresis followed by staining with Coomassie blue to visualize the bands. Lane 1, protein molecular weight markers; lanes 2 and 3, brain tissue extracts from Alzheimer’s disease (AD) patients; lanes 4 and 5, brain tissue extracts from vascular dementia (VaD) patients; lanes 6 and 7, brain tissue extracts from non-dementia (ND) control individuals and lane 8, nitrated albumin. There was 60 µg of protein loaded/well. The protein bands were excised, and tryptic digestion was performed. The peptide extracts were dried for storage, then resuspended in H_2_O/acetonitrile (98%/2%) with 0.1% formic acid prior to mass spectrometry analysis. The protein bands A, B and C, which are high molecular weight bands, were identified as non-erythrocytic spectrin-β by mass spectrometry; Band D, a 60 kDa protein band, was identified as pyruvate kinase and dihydropyrimidinase-related protein 2; Band E, an 18 kDa protein band, was peptidyl-propyl *cis-trans* isomerase; and bands F and G, the two bands which are both at about 15 kDa, were hemoglobin-α and hemoglobin-β.


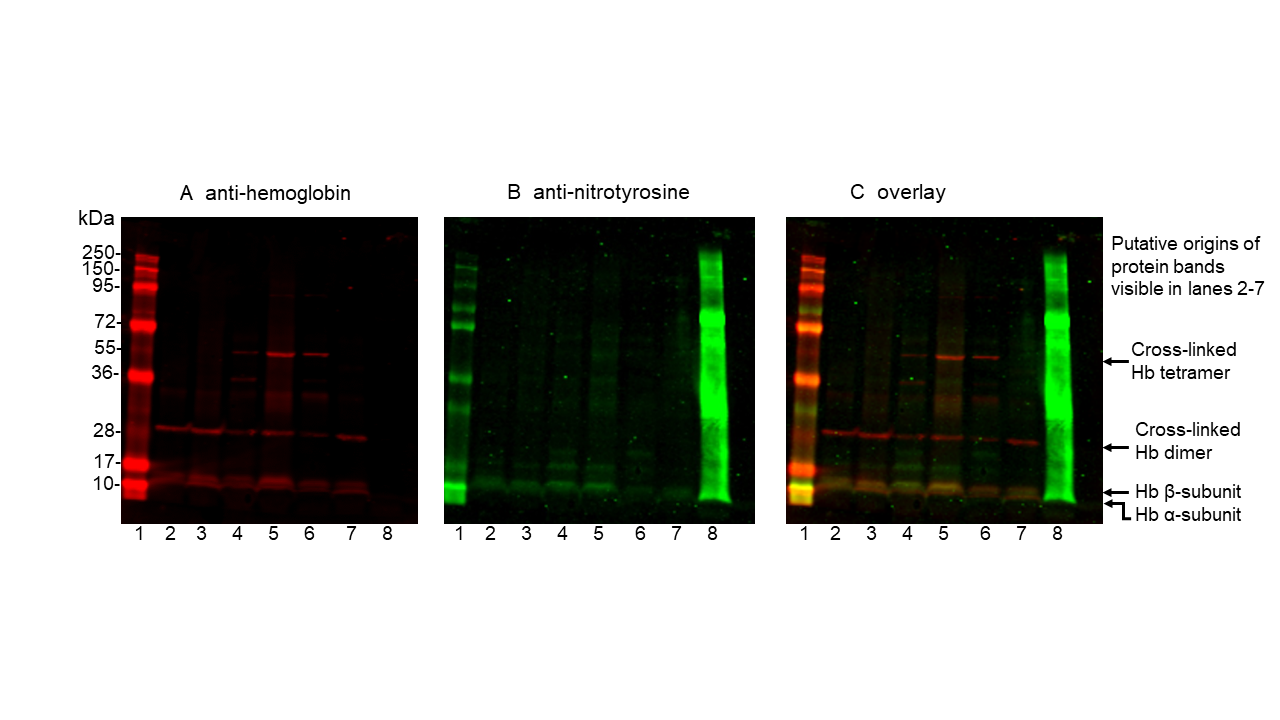


**Supplementary Fig. 2. Western blots showing total hemoglobin and nitrated hemoglobin, detected in brain tissue from Alzheimer’s disease, vascular dementia patients and non-dementia controls.** Panel A shows anti-hemoglobin staining, panel B shows anti-nitrotyrosine staining and panel C shows the colocalization of anti-hemoglobin and anti-nitrotyrosine bands (yellow color in panel C). Lane 1, protein molecular weight markers; lanes 2 and 3, brain tissue extracts from Alzheimer’s disease (AD) patients; lanes 4 and 5, brain tissue extracts from vascular dementia (VaD) patients; lanes 6 and 7, brain tissue extracts from non-dementia (ND) control individuals. There was 30 µg of protein loaded/well. These tissue extracts correspond to patients and ND control individuals who were different from those in **Fig. 1** or **Supplementary Fig. 1**. Lane 8, nitrated BSA (Enzo Life Sciences) positive control for 3-nitrotyrosine staining; there is a band at 69 kDa (corresponding to the molecular weight of BSA). There was also a second band observed at about 30 kDa possibly due to degraded albumin, either from proteolytic degradation or from chemically induced polypeptide scissions caused by ONOO^¯^ and/or associated reactive nitrogen species. The α- and β-hemoglobin monomer subunits were visible as two separate bands, with both bands having molecular weights close to 15 kDa (shown by arrows in Panel C) for lanes 2-7 inclusive, on the blot of the 8-16% gradient gel used here. The α- and β-hemoglobin monomer subunits appeared as a single band when 12% gels were used (as shown in **Fig. 1**). There were also putative hemoglobin dimers (at about 30 kDa) and tetramers (at about 55 kDa). Control blots (**Supplementary Fig. 4, Panel C**) confirmed the presence of nitrated hemoglobin multimers in human brain tissue lysates from patients with AD and ND controls. The anti-hemoglobin secondary antibody was labelled with IRDye680RD (red fluorescence) and the anti-nitrotyrosine secondary antibody was labelled with IRDye800CW (green fluorescence). The blot was imaged using the LiCor Odyssey CLx system.


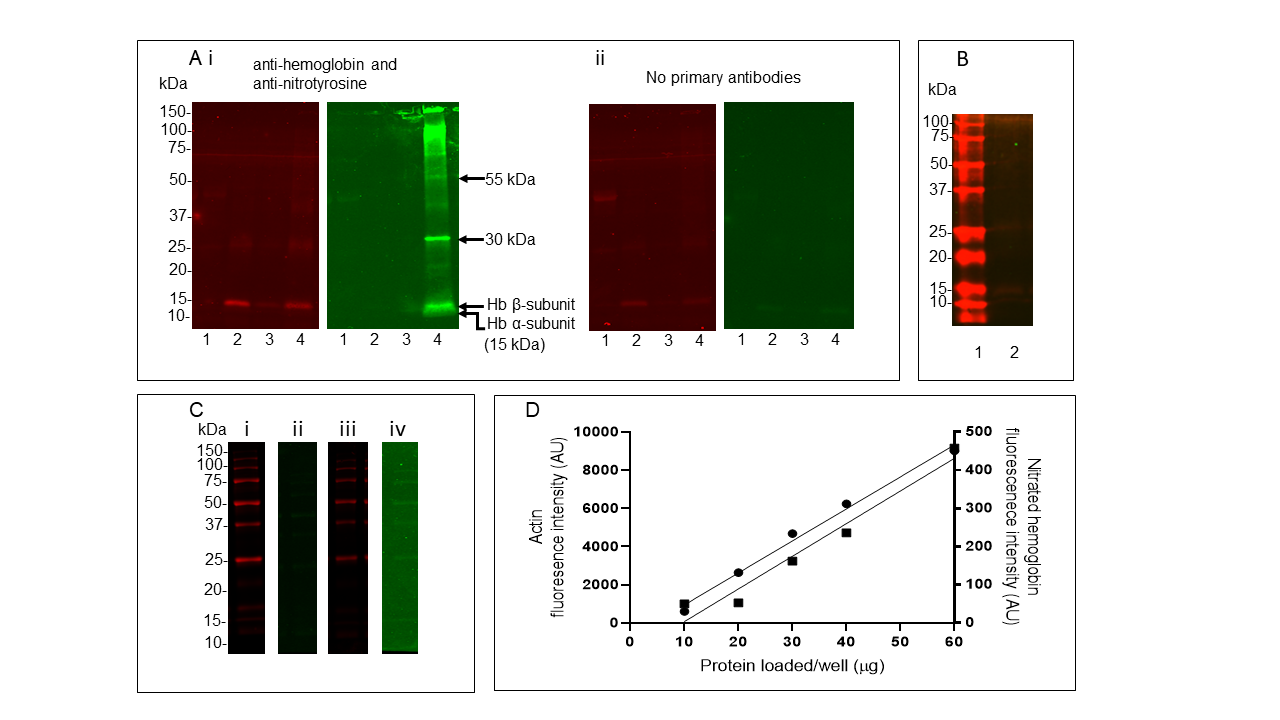


**Supplementary Fig. 3. Western blotting control experiments.** Panel A confirmed the presence of hemoglobin multimers in human brain samples, as non-specific binding of the secondary antibodies to nitrated hemoglobin did not occur. Lane 1, commercial human IgG (20 µg); lane 2, commercial human hemoglobin (10 µg); lane 3, commercial human hemoglobin (1 µg); lane 4 nitrated hemoglobin (10 µg). There was also little detectable non-specific binding of the secondary antibodies to human tissue proteins. Subpanel A (i) shows a blot which was stained with both anti-hemoglobin and anti-nitrotyrosine antibodies as well as with secondary (goat anti-rabbit IgG and goat anti-mouse IgG) antibodies (showing red and green bands). Subpanel A (ii) corresponds to the same samples incubated with secondary antibodies, but without the primary antibodies, showing an absence of staining in the green channel and minimal staining in the red channel. Panel B confirms the absence of fluorescence due to hemoglobin alone. Lane 1, molecular weight markers; and lane 2, hemoglobin (10 µg) without any primary or secondary antibodies. In Panel C, subpanels (i) and (ii) show that the molecular weight markers, without any addition of antibodies, were visible only in the red channel at 700 nm (i) and not in the green channel at 800 nm (ii); confirming that there was no spectral overlap between the red and green channels. When the blots were incubated with the anti-nitrotyrosine primary antibody and secondary antibody (iii and iv) the markers were visible in the red channel (iii) but were only very faintly visible in the green channel (iv). Panel D, the anti-actin band intensity showed a linear relationship with anti-nitrotyrosine band intensity over the dilutions of the brain homogenate sample (10-60 µg protein/well). The curve defined by the solid circles represents anti-nitrotyrosine and the curve defined by the solid squares represents anti-actin. In panel A, the molecular weight marker lane is not shown, but the molecular weight marker positions are shown to the left of the blots.


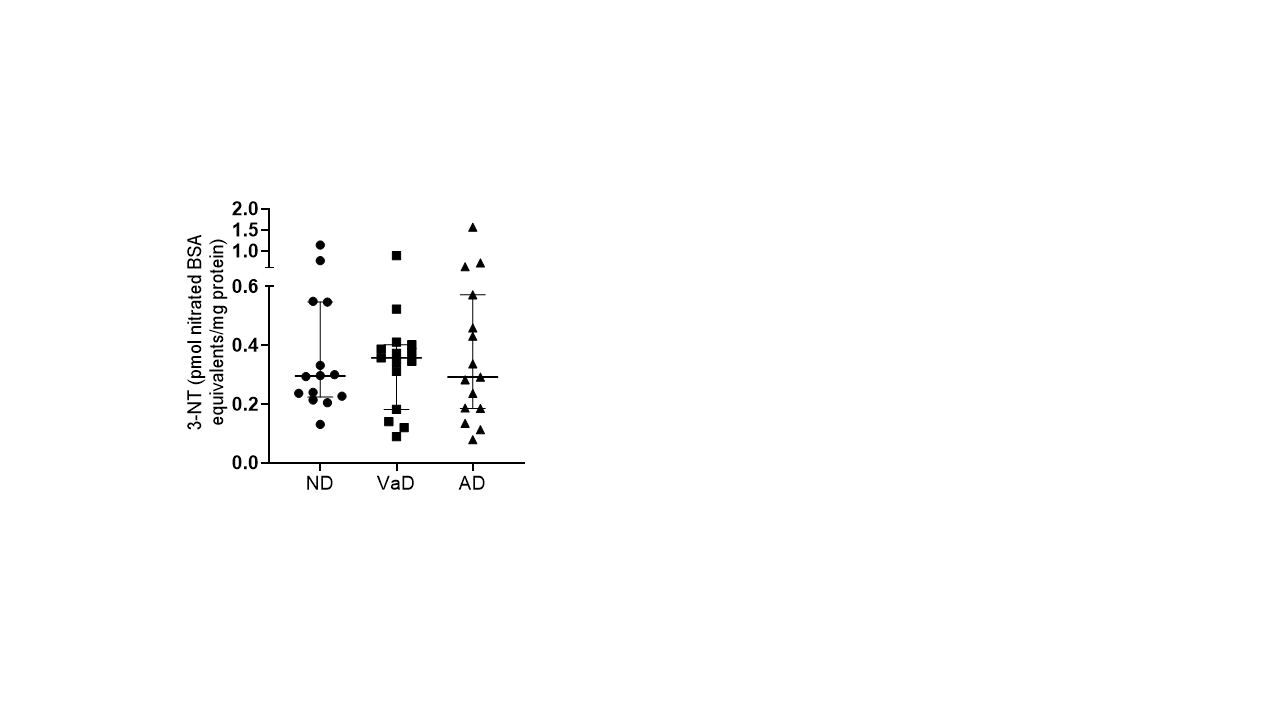


**Supplementary Fig. 4. Levels of 3-nitrotyrosine residues in the total protein extracts from the frontal lobe brain tissue homogenates from Alzheimer’s disease, vascular dementia patients and non-dementia healthy controls.** The data represent median and inter-quartile ranges for 3-nitrotyrosine in Alzheimer’s disease (AD), vascular dementia (VaD) and non-dementia (ND) control brain tissue. The total 3-nitrotyrosine within protein extracts was measured in duplicate, in n=15 patients from each of the three groups, by ECLISA as described by Knight *et al* [[56](#_ENREF_56)]. All three groups exhibited a substantial inter-individual variation in total 3-nitrotyrosine levels, and the median values showed no statistically significant differences. 3-Nitrotyrosine, 3-NT.


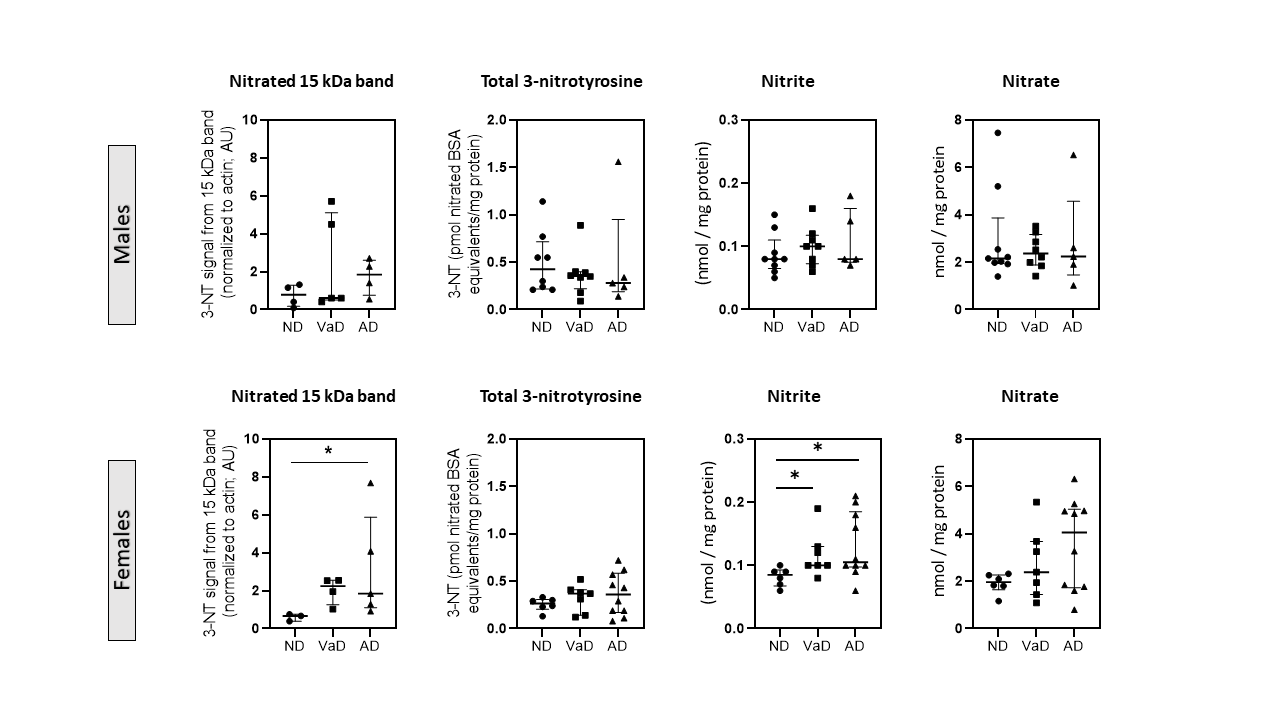


**Supplementary Fig. 5. Sub-analysis, according to sex, of the levels of protein nitration, nitrite and nitrate.** The graphs show the levels of the normalized 3-nitrotyrosine-associated fluorescence intensity of the15 kDa band (fluorescence intensity normalized to actin AU, as determined by western blotting), total nitrated protein (pmol nitrated BSA equivalents /mg protein, as determined by ECLISA) and the levels of NO_2_¯ and NO_3_¯ (nmol/mg protein, as determined by ozone-based chemiluminescence) in lysates of the frontal lobe brain tissue from patients with Alzheimer’s disease (AD), vascular dementia (VaD) or non-dementia (ND) people. In the female control tissue, the median fluorescence intensity of the nitrated 15 kDa bands was significantly higher in the AD group compared to ND controls: ND, 0.67 (0.39–0.75) AU; VaD 2.25 (1.26-2.55) AU and AD 1.85 (1.11-5.88) AU (* Mann Whitney U test, P < 0.05). Additionally, in the female controls, the median NO_2_¯ levels were significantly higher in both the VaD and AD groups compared to ND controls: ND, 0.09 (0.07–0.09) AU; VaD 0.1 (0.10-0.13) AU; AD 0.11 (0.10-0.19) AU (* Mann Whitney U test, P < 0.05).

**Reference**

1. Y. Ishihama, Y. Oda, T. Tabata, T. Sato, T. Nagasu, J. Rappsilber, M. Mann, Exponentially modified protein abundance index (emPAI) for estimation of absolute protein amount in proteomics by the number of sequenced peptides per protein, Mol Cell Proteomics. 4.(2005) 1265-72.
